# *DOCK2* is involved in the host genetics and biology of severe COVID-19

**DOI:** 10.1038/s41586-022-05163-5

**Published:** 2022-08-08

**Authors:** Ho Namkoong, Ryuya Edahiro, Tomomi Takano, Hiroshi Nishihara, Yuya Shirai, Kyuto Sonehara, Hiromu Tanaka, Shuhei Azekawa, Yohei Mikami, Ho Lee, Takanori Hasegawa, Koji Okudela, Daisuke Okuzaki, Daisuke Motooka, Masahiro Kanai, Tatsuhiko Naito, Kenichi Yamamoto, Qingbo S. Wang, Ryunosuke Saiki, Rino Ishihara, Yuta Matsubara, Junko Hamamoto, Hiroyuki Hayashi, Yukihiro Yoshimura, Natsuo Tachikawa, Emmy Yanagita, Takayoshi Hyugaji, Eigo Shimizu, Kotoe Katayama, Yasuhiro Kato, Takayoshi Morita, Kazuhisa Takahashi, Norihiro Harada, Toshio Naito, Makoto Hiki, Yasushi Matsushita, Haruhi Takagi, Ryousuke Aoki, Ai Nakamura, Sonoko Harada, Hitoshi Sasano, Hiroki Kabata, Katsunori Masaki, Hirofumi Kamata, Shinnosuke Ikemura, Shotaro Chubachi, Satoshi Okamori, Hideki Terai, Atsuho Morita, Takanori Asakura, Junichi Sasaki, Hiroshi Morisaki, Yoshifumi Uwamino, Kosaku Nanki, Sho Uchida, Shunsuke Uno, Tomoyasu Nishimura, Takashi Ishiguro, Taisuke Isono, Shun Shibata, Yuma Matsui, Chiaki Hosoda, Kenji Takano, Takashi Nishida, Yoichi Kobayashi, Yotaro Takaku, Noboru Takayanagi, Soichiro Ueda, Ai Tada, Masayoshi Miyawaki, Masaomi Yamamoto, Eriko Yoshida, Reina Hayashi, Tomoki Nagasaka, Sawako Arai, Yutaro Kaneko, Kana Sasaki, Etsuko Tagaya, Masatoshi Kawana, Ken Arimura, Kunihiko Takahashi, Tatsuhiko Anzai, Satoshi Ito, Akifumi Endo, Yuji Uchimura, Yasunari Miyazaki, Takayuki Honda, Tomoya Tateishi, Shuji Tohda, Naoya Ichimura, Kazunari Sonobe, Chihiro Tani Sassa, Jun Nakajima, Yasushi Nakano, Yukiko Nakajima, Ryusuke Anan, Ryosuke Arai, Yuko Kurihara, Yuko Harada, Kazumi Nishio, Tetsuya Ueda, Masanori Azuma, Ryuichi Saito, Toshikatsu Sado, Yoshimune Miyazaki, Ryuichi Sato, Yuki Haruta, Tadao Nagasaki, Yoshinori Yasui, Yoshinori Hasegawa, Yoshikazu Mutoh, Tomoki Kimura, Tomonori Sato, Reoto Takei, Satoshi Hagimoto, Yoichiro Noguchi, Yasuhiko Yamano, Hajime Sasano, Sho Ota, Yasushi Nakamori, Kazuhisa Yoshiya, Fukuki Saito, Tomoyuki Yoshihara, Daiki Wada, Hiromu Iwamura, Syuji Kanayama, Shuhei Maruyama, Takashi Yoshiyama, Ken Ohta, Hiroyuki Kokuto, Hideo Ogata, Yoshiaki Tanaka, Kenichi Arakawa, Masafumi Shimoda, Takeshi Osawa, Hiroki Tateno, Isano Hase, Shuichi Yoshida, Shoji Suzuki, Miki Kawada, Hirohisa Horinouchi, Fumitake Saito, Keiko Mitamura, Masao Hagihara, Junichi Ochi, Tomoyuki Uchida, Rie Baba, Daisuke Arai, Takayuki Ogura, Hidenori Takahashi, Shigehiro Hagiwara, Genta Nagao, Shunichiro Konishi, Ichiro Nakachi, Koji Murakami, Mitsuhiro Yamada, Hisatoshi Sugiura, Hirohito Sano, Shuichiro Matsumoto, Nozomu Kimura, Yoshinao Ono, Hiroaki Baba, Yusuke Suzuki, Sohei Nakayama, Keita Masuzawa, Shinichi Namba, Ken Suzuki, Yoko Naito, Yu-Chen Liu, Ayako Takuwa, Fuminori Sugihara, James B. Wing, Shuhei Sakakibara, Nobuyuki Hizawa, Takayuki Shiroyama, Satoru Miyawaki, Yusuke Kawamura, Akiyoshi Nakayama, Hirotaka Matsuo, Yuichi Maeda, Takuro Nii, Yoshimi Noda, Takayuki Niitsu, Yuichi Adachi, Takatoshi Enomoto, Saori Amiya, Reina Hara, Yuta Yamaguchi, Teruaki Murakami, Tomoki Kuge, Kinnosuke Matsumoto, Yuji Yamamoto, Makoto Yamamoto, Midori Yoneda, Toshihiro Kishikawa, Shuhei Yamada, Shuhei Kawabata, Noriyuki Kijima, Masatoshi Takagaki, Noah Sasa, Yuya Ueno, Motoyuki Suzuki, Norihiko Takemoto, Hirotaka Eguchi, Takahito Fukusumi, Takao Imai, Munehisa Fukushima, Haruhiko Kishima, Hidenori Inohara, Kazunori Tomono, Kazuto Kato, Meiko Takahashi, Fumihiko Matsuda, Haruhiko Hirata, Yoshito Takeda, Hidefumi Koh, Tadashi Manabe, Yohei Funatsu, Fumimaro Ito, Takahiro Fukui, Keisuke Shinozuka, Sumiko Kohashi, Masatoshi Miyazaki, Tomohisa Shoko, Mitsuaki Kojima, Tomohiro Adachi, Motonao Ishikawa, Kenichiro Takahashi, Takashi Inoue, Toshiyuki Hirano, Keigo Kobayashi, Hatsuyo Takaoka, Kazuyoshi Watanabe, Naoki Miyazawa, Yasuhiro Kimura, Reiko Sado, Hideyasu Sugimoto, Akane Kamiya, Naota Kuwahara, Akiko Fujiwara, Tomohiro Matsunaga, Yoko Sato, Takenori Okada, Yoshihiro Hirai, Hidetoshi Kawashima, Atsuya Narita, Kazuki Niwa, Yoshiyuki Sekikawa, Koichi Nishi, Masaru Nishitsuji, Mayuko Tani, Junya Suzuki, Hiroki Nakatsumi, Takashi Ogura, Hideya Kitamura, Eri Hagiwara, Kota Murohashi, Hiroko Okabayashi, Takao Mochimaru, Shigenari Nukaga, Ryosuke Satomi, Yoshitaka Oyamada, Nobuaki Mori, Tomoya Baba, Yasutaka Fukui, Mitsuru Odate, Shuko Mashimo, Yasushi Makino, Kazuma Yagi, Mizuha Hashiguchi, Junko Kagyo, Tetsuya Shiomi, Satoshi Fuke, Hiroshi Saito, Tomoya Tsuchida, Shigeki Fujitani, Mumon Takita, Daiki Morikawa, Toru Yoshida, Takehiro Izumo, Minoru Inomata, Naoyuki Kuse, Nobuyasu Awano, Mari Tone, Akihiro Ito, Yoshihiko Nakamura, Kota Hoshino, Junichi Maruyama, Hiroyasu Ishikura, Tohru Takata, Toshio Odani, Masaru Amishima, Takeshi Hattori, Yasuo Shichinohe, Takashi Kagaya, Toshiyuki Kita, Kazuhide Ohta, Satoru Sakagami, Kiyoshi Koshida, Kentaro Hayashi, Tetsuo Shimizu, Yutaka Kozu, Hisato Hiranuma, Yasuhiro Gon, Namiki Izumi, Kaoru Nagata, Ken Ueda, Reiko Taki, Satoko Hanada, Kodai Kawamura, Kazuya Ichikado, Kenta Nishiyama, Hiroyuki Muranaka, Kazunori Nakamura, Naozumi Hashimoto, Keiko Wakahara, Koji Sakamoto, Norihito Omote, Akira Ando, Nobuhiro Kodama, Yasunari Kaneyama, Shunsuke Maeda, Takashige Kuraki, Takemasa Matsumoto, Koutaro Yokote, Taka-Aki Nakada, Ryuzo Abe, Taku Oshima, Tadanaga Shimada, Masahiro Harada, Takeshi Takahashi, Hiroshi Ono, Toshihiro Sakurai, Takayuki Shibusawa, Yoshifumi Kimizuka, Akihiko Kawana, Tomoya Sano, Chie Watanabe, Ryohei Suematsu, Hisako Sageshima, Ayumi Yoshifuji, Kazuto Ito, Saeko Takahashi, Kota Ishioka, Morio Nakamura, Makoto Masuda, Aya Wakabayashi, Hiroki Watanabe, Suguru Ueda, Masanori Nishikawa, Yusuke Chihara, Mayumi Takeuchi, Keisuke Onoi, Jun Shinozuka, Atsushi Sueyoshi, Yoji Nagasaki, Masaki Okamoto, Sayoko Ishihara, Masatoshi Shimo, Yoshihisa Tokunaga, Yu Kusaka, Takehiko Ohba, Susumu Isogai, Aki Ogawa, Takuya Inoue, Satoru Fukuyama, Yoshihiro Eriguchi, Akiko Yonekawa, Keiko Kan-o, Koichiro Matsumoto, Kensuke Kanaoka, Shoichi Ihara, Kiyoshi Komuta, Yoshiaki Inoue, Shigeru Chiba, Kunihiro Yamagata, Yuji Hiramatsu, Hirayasu Kai, Koichiro Asano, Tsuyoshi Oguma, Yoko Ito, Satoru Hashimoto, Masaki Yamasaki, Yu Kasamatsu, Yuko Komase, Naoya Hida, Takahiro Tsuburai, Baku Oyama, Minoru Takada, Hidenori Kanda, Yuichiro Kitagawa, Tetsuya Fukuta, Takahito Miyake, Shozo Yoshida, Shinji Ogura, Shinji Abe, Yuta Kono, Yuki Togashi, Hiroyuki Takoi, Ryota Kikuchi, Shinichi Ogawa, Tomouki Ogata, Shoichiro Ishihara, Arihiko Kanehiro, Shinji Ozaki, Yasuko Fuchimoto, Sae Wada, Nobukazu Fujimoto, Kei Nishiyama, Mariko Terashima, Satoru Beppu, Kosuke Yoshida, Osamu Narumoto, Hideaki Nagai, Nobuharu Ooshima, Mitsuru Motegi, Akira Umeda, Kazuya Miyagawa, Hisato Shimada, Mayu Endo, Yoshiyuki Ohira, Masafumi Watanabe, Sumito Inoue, Akira Igarashi, Masamichi Sato, Hironori Sagara, Akihiko Tanaka, Shin Ohta, Tomoyuki Kimura, Yoko Shibata, Yoshinori Tanino, Takefumi Nikaido, Hiroyuki Minemura, Yuki Sato, Yuichiro Yamada, Takuya Hashino, Masato Shinoki, Hajime Iwagoe, Hiroshi Takahashi, Kazuhiko Fujii, Hiroto Kishi, Masayuki Kanai, Tomonori Imamura, Tatsuya Yamashita, Masakiyo Yatomi, Toshitaka Maeno, Shinichi Hayashi, Mai Takahashi, Mizuki Kuramochi, Isamu Kamimaki, Yoshiteru Tominaga, Tomoo Ishii, Mitsuyoshi Utsugi, Akihiro Ono, Toru Tanaka, Takeru Kashiwada, Kazue Fujita, Yoshinobu Saito, Masahiro Seike, Hiroko Watanabe, Hiroto Matsuse, Norio Kodaka, Chihiro Nakano, Takeshi Oshio, Takatomo Hirouchi, Shohei Makino, Moritoki Egi, The Biobank Japan Project, Koichi Matsuda, Yuji Yamanashi, Yoichi Furukawa, Takayuki Morisaki, Yoshinori Murakami, Yoichiro Kamatani, Kaori Muto, Akiko Nagai, Wataru Obara, Ken Yamaji, Kazuhisa Takahashi, Satoshi Asai, Yasuo Takahashi, Takao Suzuki, Nobuaki Sinozaki, Hiroki Yamaguchi, Shiro Minami, Shigeo Murayama, Kozo Yoshimori, Satoshi Nagayama, Daisuke Obata, Masahiko Higashiyama, Akihide Masumoto, Yukihiro Koretsune, Yosuke Omae, Yasuhito Nannya, Takafumi Ueno, Kazuhiko Katayama, Masumi Ai, Yoshinori Fukui, Atsushi Kumanogoh, Toshiro Sato, Naoki Hasegawa, Katsushi Tokunaga, Makoto Ishii, Ryuji Koike, Yuko Kitagawa, Akinori Kimura, Seiya Imoto, Satoru Miyano, Seishi Ogawa, Takanori Kanai, Koichi Fukunaga, Yukinori Okada

**Affiliations:** 1 Department of Infectious Diseases, Keio University School of Medicine, Tokyo, Japan; 2 Department of Statistical Genetics, Osaka University Graduate School of Medicine, Suita, Japan; 3 Department of Respiratory Medicine and Clinical Immunology, Osaka University Graduate School of Medicine, Suita, Japan; 4 Laboratory of Veterinary Infectious Disease, School of Veterinary Medicine, Kitasato University, Aomori, Japan; 5 Genomics Unit, Keio Cancer Center, Keio University Hospital, Tokyo, Japan; 6 Integrated Frontier Research for Medical Science Division, Institute for Open and Transdisciplinary Research Initiatives, Osaka University, Suita, Japan; 7 Division of Pulmonary Medicine, Department of Medicine, Keio University School of Medicine, Tokyo, Japan; 8 Division of Gastroenterology and Hepatology, Department of Medicine, Keio University School of Medicine, Tokyo, Japan; 9 M&D Data Science Center, Tokyo Medical and Dental University, Tokyo, Japan; 10 Department of Pathology, Graduate School of Medicine, Yokohama City University, Yokohama, Japan; 11 Single Cell Genomics, Human Immunology, WPI Immunology Frontier Research Center, Osaka University, Suita, Japan; 12 Genome Information Research Center, Research Institute for Microbial Diseases, Osaka University, Suita, Japan; 13 Department of Biomedical Informatics, Harvard Medical School, Boston, MA USA; 14 Department of Pathology and Tumor Biology, Kyoto University, Kyoto, Japan; 15 Division of Pathology, Yokohama Municipal Citizen’s Hospital, Yokohama, Japan; 16 Division of Infectious Disease, Yokohama Municipal Citizen’s Hospital, Yokohama, Japan; 17 Division of Health Medical Intelligence, Human Genome Center, the Institute of Medical Science, the University of Tokyo, Tokyo, Japan; 18 Department of Immunopathology, Immunology Frontier Research Center (WPI-IFReC), Osaka University, Suita, Japan; 19 Department of Respiratory Medicine, Juntendo University Faculty of Medicine and Graduate School of Medicine, Tokyo, Japan; 20 Department of General Medicine, Juntendo University Faculty of Medicine and Graduate School of Medicine, Tokyo, Japan; 21 Department of Emergency and Disaster Medicine, Juntendo University Faculty of Medicine and Graduate School of Medicine, Tokyo, Japan; 22 Department of Cardiovascular Biology and Medicine, Juntendo University Faculty of Medicine and Graduate School of Medicine, Tokyo, Japan; 23 Department of Internal Medicine and Rheumatology, Juntendo University Faculty of Medicine and Graduate School of Medicine, Tokyo, Japan; 24 Department of Nephrology, Juntendo University Faculty of Medicine and Graduate School of Medicine, Tokyo, Japan; 25 Atopy (Allergy) Research Center, Juntendo University Graduate School of Medicine, Tokyo, Japan; 26 Department of Emergency and Critical Care Medicine, Keio University School of Medicine, Tokyo, Japan; 27 Department of Anesthesiology, Keio University School of Medicine, Tokyo, Japan; 28 Department of Laboratory Medicine, Keio University School of Medicine, Tokyo, Japan; 29 Keio University Health Center, Tokyo, Japan; 30 Department of Respiratory Medicine, Saitama Cardiovascular and Respiratory Center, Kumagaya, Japan; 31 JCHO (Japan Community Health care Organization) Saitama Medical Center, Internal Medicine, Saitama, Japan; 32 Department of Respiratory Medicine, Tokyo Women’s Medical University, Tokyo, Japan; 33 Department of General Medicine, Tokyo Women’s Medical University, Tokyo, Japan; 34 Clinical Research Center, Tokyo Medical and Dental University Hospital of Medicine, Tokyo, Japan; 35 Department of Medical Informatics, Tokyo Medical and Dental University Hospital of Medicine, Tokyo, Japan; 36 Respiratory Medicine, Tokyo Medical and Dental University, Tokyo, Japan; 37 Clinical Laboratory, Tokyo Medical and Dental University Hospital of Medicine, Tokyo, Japan; 38 Kawasaki Municipal Ida Hospital, Department of Internal Medicine, Kawasaki, Japan; 39 Department of Respiratory Medicine, Osaka Saiseikai Nakatsu Hospital, Osaka, Japan; 40 Department of Infection Control, Osaka Saiseikai Nakatsu Hospital, Osaka, Japan; 41 Department of Infectious Diseases, Tosei General Hospital, Seto, Japan; 42 Department of Respiratory Medicine and Allergy, Tosei General Hospital, Seto, Japan; 43 Department of Emergency and Critical Care Medicine, Kansai Medical University General Medical Center, Moriguchi, Japan; 44 Fukujuji hospital, Kiyose, Japan; 45 Department of Pulmonary Medicine, Saitama City Hospital, Saitama, Japan; 46 Department of Infectious Diseases, Saitama City Hospital, Saitama, Japan; 47 Department of General Thoracic Surgery, Saitama City Hospital, Saitama, Japan; 48 Department of Pulmonary Medicine, Eiju General Hospital, Tokyo, Japan; 49 Division of Infection Control, Eiju General Hospital, Tokyo, Japan; 50 Department of Hematology, Eiju General Hospital, Tokyo, Japan; 51 Saiseikai Utsunomiya Hospital, Utsunomiya, Japan; 52 Department of Respiratory Medicine, Tohoku University Graduate School of Medicine, Sendai, Japan; 53 Department of Infectious Diseases, Tohoku University Graduate School of Medicine, Sendai, Japan; 54 Department of Respiratory Medicine, Kitasato University Kitasato Institute Hospital, Tokyo, Japan; 55 Core Instrumentation Facility, Immunology Frontier Research Center and Research Institute for Microbial Diseases, Osaka University, Suita, Japan; 56 Laboratory of Human Immunology (Single Cell Immunology), Immunology Frontier Research Center, Osaka University, Suita, Japan; 57 Center for Infectious Disease Education and Research (CiDER), Osaka University, Suita, Japan; 58 Laboratory of Immune Regulation, Immunology Frontier Research Center, Osaka University, Suita, Japan; 59 Department of Pulmonary Medicine, Faculty of Medicine, University of Tsukuba, Tsukuba, Japan; 60 Department of Neurosurgery, Faculty of Medicine, the University of Tokyo, Tokyo, Japan; 61 Department of Integrative Physiology and Bio-Nano Medicine, National Defense Medical College, Tokorozawa, Japan; 62 Department of Otorhinolaryngology-Head and Neck Surgery, Osaka University Graduate School of Medicine, Suita, Japan; 63 Department of Head and Neck Surgery, Aichi Cancer Center Hospital, Nagoya, Japan; 64 Department of Neurosurgery, Osaka University Graduate School of Medicine, Suita, Japan; 65 Department of Otolaryngology and Head and Neck Surgery, Kansai Rosai Hospital, Hyogo, Japan; 66 Division of Infection Control and Prevention, Osaka University Hospital, Suita, Japan; 67 Department of Biomedical Ethics and Public Policy, Osaka University Graduate School of Medicine, Suita, Japan; 68 Center for Genomic Medicine, Kyoto University Graduate School of Medicine, Kyoto, Japan; 69 Tachikawa Hospital, Tachikawa, Japan; 70 Department of Emergency and Critical Care Medicine, Tokyo Women’s Medical University Medical Center East, Tokyo, Japan; 71 Department of Medicine, Tokyo Women’s Medical University Medical Center East, Tokyo, Japan; 72 Department of Pediatrics, Tokyo Women’s Medical University Medical Center East, Tokyo, Japan; 73 Internal Medicine, Sano Kosei General Hospital, Sano, Japan; 74 Japan Community Health Care Organization, Kanazawa Hospital, Kanazawa, Japan; 75 Department of Respiratory Medicine, Saiseikai Yokohamashi Nanbu Hospital, Yokohama, Japan; 76 Department of Clinical Laboratory, Saiseikai Yokohamashi Nanbu Hospital, Yokohama, Japan; 77 Internal Medicine, Internal Medicine Center, Showa University Koto Toyosu Hospital, Tokyo, Japan; 78 Department of Respiratory Medicine, Japan Organization of Occupational Health and Safety, Kanto Rosai Hospital, Kawasaki, Japan; 79 Department of General Internal Medicine, Japan Organization of Occupational Health and Safety, Kanto Rosai Hospital, Kawasaki, Japan; 80 Ishikawa Prefectural Central Hospital, Kanazawa, Japan; 81 Kanagawa Cardiovascular and Respiratory Center, Yokohama, Japan; 82 Department of Respiratory Medicine, National Hospital Organization Tokyo Medical Center, Tokyo, Japan; 83 Department of Allergy, National Hospital Organization Tokyo Medical Center, Tokyo, Japan; 84 Department of General Internal Medicine and Infectious Diseases, National Hospital Organization Tokyo Medical Center, Tokyo, Japan; 85 Department of Respiratory Medicine, Toyohashi Municipal Hospital, Toyohashi, Japan; 86 Keiyu Hospital, Yokohama, Japan; 87 Department of Respiratory Medicine, KKR Sapporo Medical Center, Sapporo, Japan; 88 Division of General Internal Medicine, Department of Internal Medicine, St Marianna University School of Medicine, Kawasaki, Japan; 89 Department of Emergency and Critical Care Medicine, St Marianna University School of Medicine, Kawasaki, Japan; 90 Japanese Red Cross Medical Center, Tokyo, Japan; 91 Matsumoto City Hospital, Matsumoto, Japan; 92 Department of Emergency and Critical Care Medicine, Faculty of Medicine, Fukuoka University, Fukuoka, Japan; 93 Department of Infection Control, Fukuoka University Hospital, Fukuoka, Japan; 94 Department of Rheumatology, National Hospital Organization Hokkaido Medical Center, Sapporo, Japan; 95 Department of Respiratory Medicine, National Hospital Organization Hokkaido Medical Center, Sapporo, Japan; 96 Department of Emergency and Critical Care Medicine, National Hospital Organization Hokkaido Medical Center, Sapporo, Japan; 97 National Hospital Organization Kanazawa Medical Center, Kanazawa, Japan; 98 Nihon University School of Medicine, Department of Internal Medicine, Division of Respiratory Medicine, Tokyo, Japan; 99 Musashino Red Cross Hospital, Musashino, Japan; 100 Division of Respiratory Medicine, Social Welfare Organization Saiseikai Imperial Gift Foundation, Inc., Saiseikai Kumamoto Hospital, Kumamoto, Japan; 101 Department of Respiratory Medicine, Nagoya University Graduate School of Medicine, Nagoya, Japan; 102 Department of Internal Medicine, Fukuoka Tokushukai Hospital, Kasuga, Japan; 103 Respiratory Medicine, Fukuoka Tokushukai Hospital, Kasuga, Japan; 104 Department of Endocrinology, Hematology and Gerontology, Chiba University Graduate School of Medicine, Chiba, Japan; 105 Department of Emergency and Critical Care Medicine, Chiba University Graduate School of Medicine, Chiba, Japan; 106 National Hospital Organization Kumamoto Medical Center, Kumamoto, Japan; 107 Division of Infectious Diseases and Respiratory Medicine, Department of Internal Medicine, National Defense Medical College, Tokorozawa, Japan; 108 Sapporo City General Hospital, Sapporo, Japan; 109 Department of Internal Medicine, Tokyo Saiseikai Central Hospital, Tokyo, Japan; 110 Department of Pulmonary Medicine, Tokyo Saiseikai Central Hospital, Tokyo, Japan; 111 Department of Respiratory Medicine, Fujisawa City Hospital, Fujisawa, Japan; 112 Uji-Tokushukai Medical Center, Uji, Japan; 113 Department of Infectious Disease and Clinical Research Institute, National Hospital Organization Kyushu Medical Center, Fukuoka, Japan; 114 Department of Respirology, National Hospital Organization Kyushu Medical Center, Fukuoka, Japan; 115 Division of Respirology, Rheumatology, and Neurology, Department of Internal Medicine, Kurume University School of Medicine, Kurume, Japan; 116 Department of Infectious Disease, National Hospital Organization Kyushu Medical Center, Fukuoka, Japan; 117 Ome Municipal General Hospital, Ome, Japan; 118 Research Institute for Diseases of the Chest, Graduate School of Medical Sciences, Kyushu University, Fukuoka, Japan; 119 Department of Medicine and Biosystemic Science, Kyushu University Graduate School of Medical Sciences, Fukuoka, Japan; 120 Daini Osaka Police Hospital, Osaka, Japan; 121 Department of Emergency and Critical Care Medicine, Faculty of Medicine, University of Tsukuba, Tsukuba, Japan; 122 Department of Hematology, Faculty of Medicine, University of Tsukuba, Tsukuba, Japan; 123 Department of Nephrology, Faculty of Medicine, University of Tsukuba, Tsukuba, Japan; 124 Department of Cardiovascular Surgery, Faculty of Medicine, University of Tsukuba, Tsukuba, Japan; 125 Division of Pulmonary Medicine, Department of Medicine, Tokai University School of Medicine, Isehara, Japan; 126 Department of Anesthesiology and Intensive Care Medicine, Kyoto Prefectural University of Medicine, Kyoto, Japan; 127 Department of Infection Control and Laboratory Medicine, Kyoto Prefectural University of Medicine, Kyoto, Japan; 128 Department of Respiratory Internal Medicine, St Marianna University School of Medicine, Yokohama Seibu Hospital, Yokohama, Japan; 129 KINSHUKAI Hanwa, The Second Hospital, Osaka, Japan; 130 Gifu University School of Medicine Graduate School of Medicine, Emergency and Disaster Medicine, Gifu, Japan; 131 Department of Respiratory Medicine, Tokyo Medical University Hospital, Tokyo, Japan; 132 JA Toride Medical Hospital, Toride, Japan; 133 Okayama Rosai Hospital, Okayama, Japan; 134 Himeji St Mary’s Hospital, Himeji, Japan; 135 Emergency and Critical Care, Niigata University, Niigata, Japan; 136 Emergency and Critical Care Center, National Hospital Organization Kyoto Medical Center, Kyoto, Japan; 137 National Hospital Organization Tokyo Hospital Hospital, Kiyose, Japan; 138 Fujioka General Hospital, Fujioka, Japan; 139 Department of General Medicine, School of Medicine, International University of Health and Welfare Shioya Hospital, Ohtawara, Japan; 140 Department of Pharmacology, School of Pharmacy, International University of Health and Welfare, Ohtawara, Japan; 141 Department of Respiratory Medicine, International University of Health and Welfare Shioya Hospital, Ohtawara, Japan; 142 Department of Clinical Laboratory, International University of Health and Welfare Shioya Hospital, Ohtawara, Japan; 143 Department of Cardiology, Pulmonology, and Nephrology, Yamagata University Faculty of Medicine, Yamagata, Japan; 144 Division of Respiratory Medicine and Allergology, Department of Medicine, School of Medicine, Showa University, Tokyo, Japan; 145 Department of Pulmonary Medicine, Fukushima Medical University, Fukushima, Japan; 146 Kansai Electric Power Hospital, Osaka, Japan; 147 Department of Infectious Diseases, Kumamoto City Hospital, Kumamoto, Japan; 148 Department of Respiratory Medicine, Kumamoto City Hospital, Kumamoto, Japan; 149 Department of Emergency and Critical Care Medicine, Tokyo Metropolitan Police Hospital, Tokyo, Japan; 150 Department of Respiratory Medicine, Gunma University Graduate School of Medicine, Maebashi, Japan; 151 National Hospital Organization Saitama Hospital, Wako, Japan; 152 Tokyo Medical University Ibaraki Medical Center, Inashiki, Japan; 153 Department of Internal Medicine, Kiryu Kosei General Hospital, Kiryu, Japan; 154 Department of Pulmonary Medicine and Oncology, Graduate School of Medicine, Nippon Medical School, Tokyo, Japan; 155 Division of Respiratory Medicine, Tsukuba Kinen General Hospital, Tsukuba, Japan; 156 Division of Respiratory Medicine, Department of Internal Medicine, Toho University Ohashi Medical Center, Tokyo, Japan; 157 Division of Anesthesiology, Department of Surgery Related, Kobe University Graduate School of Medicine, Kobe, Japan; 158 Genome Medical Science Project (Toyama), National Center for Global Health and Medicine, Tokyo, Japan; 159 School of Life Science and Technology, Tokyo Institute of Technology, Tokyo, Japan; 160 Laboratory of Viral Infection, Department of Infection Control and Immunology, Ōmura Satoshi Memorial Institute and Graduate School of Infection Control Sciences, Kitasato University, Tokyo, Japan; 161 Department of Insured Medical Care Management, Tokyo Medical and Dental University Hospital of Medicine, Tokyo, Japan; 162 Division of Immunogenetics, Department of Immunobiology and Neuroscience, Medical Institute of Bioregulation, Kyushu University, Fukuoka, Japan; 163 Department of Organoid Medicine, Keio University School of Medicine, Tokyo, Japan; 164 Medical Innovation Promotion Center, Tokyo Medical and Dental University, Tokyo, Japan; 165 Department of Surgery, Keio University School of Medicine, Tokyo, Japan; 166 Institute of Research, Tokyo Medical and Dental University, Tokyo, Japan; 167 Institute for the Advanced Study of Human Biology (WPI-ASHBi), Kyoto University, Kyoto, Japan; 168 Department of Medicine, Center for Hematology and Regenerative Medicine, Karolinska Institute, Stockholm, Sweden; 169 AMED-CREST, Japan Agency for Medical Research and Development, Tokyo, Japan; 170 Laboratory for Systems Genetics, RIKEN Center for Integrative Medical Sciences, Yokohama, Japan; 171 Laboratory of Statistical Immunology, Immunology Frontier Research Center (WPI-IFReC), Osaka University, Suita, Japan; 172 Department of Genome Informatics, Graduate School of Medicine, the University of Tokyo, Tokyo, Japan; 173 Laboratory of Genome Technology, Human Genome Center, Institute of Medical Science, The University of Tokyo, Tokyo, Japan; 174 Laboratory of Clinical Genome Sequencing, Graduate School of Frontier Sciences, The University of Tokyo, Tokyo, Japan; 175 Division of Genetics, The Institute of Medical Science, The University of Tokyo, Tokyo, Japan; 176 Division of Clinical Genome Research, Institute of Medical Science, The University of Tokyo, Tokyo, Japan; 177 Division of Molecular Pathology, IMSUT Hospital Department of Internal Medicine, Institute of Medical Science, The University of Tokyo, Tokyo, Japan; 178 Department of Cancer Biology, Institute of Medical Science, The University of Tokyo, Tokyo, Japan; 179 Laboratory of Complex Trait Genomics, Graduate School of Frontier Sciences, The University of Tokyo, Tokyo, Japan; 180 Department of Public Policy, Institute of Medical Science, The University of Tokyo, Tokyo, Japan; 181 Department of Urology, Iwate Medical University, Iwate, Japan; 182 Department of Internal Medicine and Rheumatology, Juntendo University Graduate School of Medicine, Tokyo, Japan; 183 Division of Pharmacology, Department of Biomedical Science, Nihon University School of Medicine, Tokyo, Japan; 184 Division of Genomic Epidemiology and Clinical Trials, Clinical Trials Research Center, Nihon University School of Medicine, Tokyo, Japan; 185 Tokushukai Group, Tokyo, Japan; 186 Departmentof Hematology, Nippon Medical School, Tokyo, Japan; 187 Department of Bioregulation, Nippon Medical School, Kawasaki, Japan; 188 Tokyo Metropolitan Geriatric Hospital and Institute of Gerontology, Tokyo, Japan; 189 Fukujuji Hospital, Japan Anti-Tuberculosis Association, Tokyo, Japan; 190 The Cancer Institute Hospital of the Japanese Foundation for Cancer Research, Tokyo, Japan; 191 Center for Clinical Research and Advanced Medicine, Shiga University of Medical Science, Shiga, Japan; 192 Department of General Thoracic Surgery, Osaka International Cancer Institute, Osaka, Japan; 193 Iizuka Hospital, Fukuoka, Japan; 194 National Hospital Organization Osaka National Hospital, Osaka, Japan

**Keywords:** Genome-wide association studies, Genetics research, SARS-CoV-2, Viral infection, Immunogenetics

## Abstract

Identifying the host genetic factors underlying severe COVID-19 is an emerging challenge^1–5^. Here we conducted a genome-wide association study (GWAS) involving 2,393 cases of COVID-19 in a cohort of Japanese individuals collected during the initial waves of the pandemic, with 3,289 unaffected controls. We identified a variant on chromosome 5 at 5q35 (rs60200309-A), close to the dedicator of cytokinesis 2 gene (*DOCK2*), which was associated with severe COVID-19 in patients less than 65 years of age. This risk allele was prevalent in East Asian individuals but rare in Europeans, highlighting the value of genome-wide association studies in non-European populations. RNA-sequencing analysis of 473 bulk peripheral blood samples identified decreased expression of *DOCK2* associated with the risk allele in these younger patients. *DOCK2* expression was suppressed in patients with severe cases of COVID-19. Single-cell RNA-sequencing analysis (*n* = 61 individuals) identified cell-type-specific downregulation of *DOCK2* and a COVID-19-specific decreasing effect of the risk allele on *DOCK2* expression in non-classical monocytes. Immunohistochemistry of lung specimens from patients with severe COVID-19 pneumonia showed suppressed DOCK2 expression. Moreover, inhibition of DOCK2 function with CPYPP increased the severity of pneumonia in a Syrian hamster model of SARS-CoV-2 infection, characterized by weight loss, lung oedema, enhanced viral loads, impaired macrophage recruitment and dysregulated type I interferon responses. We conclude that DOCK2 has an important role in the host immune response to SARS-CoV-2 infection and the development of severe COVID-19, and could be further explored as a potential biomarker and/or therapeutic target.

## Abstract (web summary)

A genome-wide association study highlights a variant in *DOCK2*, which is common in East Asian populations but rare in Europeans, as a host genetic risk factor for severe COVID-19.

## Main

COVID-19, caused by SARS-CoV-2, remains a serious global public health issue^6^. Although promising vaccines have recently become available, the emergence of SARS-CoV-2 variants may delay the end of this pandemic^7^. COVID-19 manifests as a range of clinical presentation from asymptomatic infection to fatal respiratory or multi-organ failure, with multiple risk factors^8,9^.

The human genetic background influences the susceptibility to and/or the severity of infectious diseases. The Severe Covid-19 Genome-Wide Association Study (GWAS) Group reported a variant of *LZTFL1* at locus 3p21 with severely increased COVID-19 risk in a European population^1^. Of note, these variants demonstrated globally heterogeneous allele frequency spectra and were rarely present in East Asian people^2^.

Further GWAS efforts, including COVID-19 Human Genome Initiatives (HGI), have nominated host susceptibility genes^3–5^. However, the vast majority of existing studies have been carried out on European populations. Considering the global diversity of COVID-19 severity, COVID-19 host genetic analysis in non-European people should provide novel insights.

**Extended Data Fig. 1:**
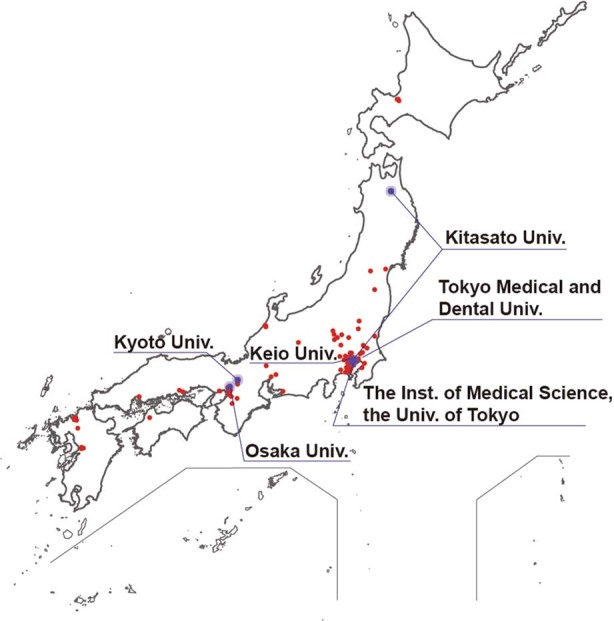
Japan COVID-19 Task Force. Japan COVID-19 Task Force is a nation-wide consortium to overcome COVID-19 pandemic in Japan, which was established in early 2020. Japan COVID-19 Task Force consists of > 100 hospitals (red dots) led by core academic institutes (blue labels), and collected DNA, RNA, and plasma from the COVID-19 cases along with detailed clinical information. The figure was originally created using sf and ggplot2 R packages based on Global Map Japan version 2.1 Vector data (Geospatial Information Authority of Japan).

The Japan COVID-19 Task Force (JCTF) was established in early 2020 as a nationwide multicentre consortium to overcome the COVID-19 pandemic (Extended Data Fig. 1 and Supplementary Table 1). Here we report the result of a large-scale GWAS of COVID-19 in Japanese individuals with systemic comparisons to results from Europeans, which identified a population-specific risk allele at the *DOCK2* region that confers a risk of severe COVID-19, particularly in individuals below 65 years of age (hereafter referred to as ‘young’). We further conducted bulk and single-cell transcriptomics, and immunohistochemical assays of the patients as well as in vivo perturbation of DOCK2 function in an animal model. We found that *DOCK2* suppression is associated with the development of severe COVID-19 in a Syrian hamster model of SARS-CoV-2 infection, and that DOCK2-mediated signalling has a key role in the host immune response to SARS-CoV-2 infection.

## Overview of the study participants

**Extended Data Fig. 2:**
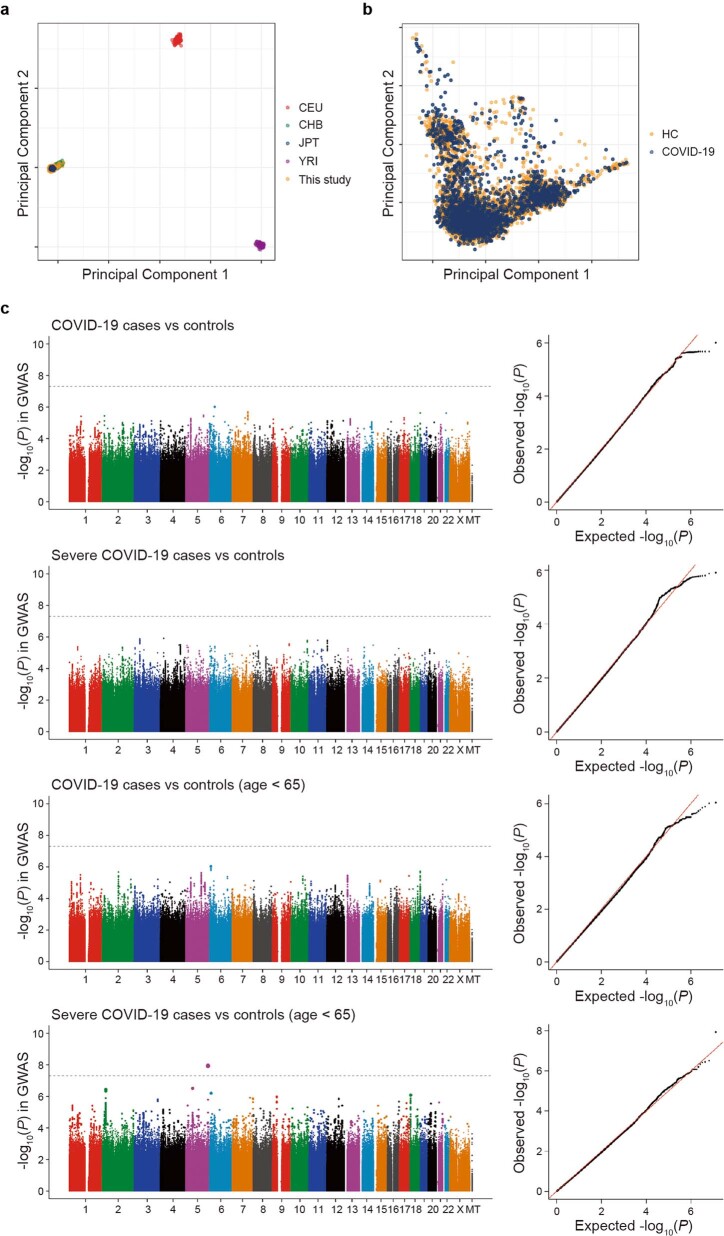
A principal component analysis plot of the GWAS participants and Manhattan and quantile-quantile plots of the GWAS. (**a**, **b**) A principal component analysis (PCA) plot of the GWAS participants (COVID-19 cases and controls) along with and without International HapMap populations (**a** and **b**, respectively). (**c**) Manhattan plots and quantile-quantile plots of the Japanese GWAS of COVID-19. Uncorrected *P* values from GWAS analysis are shown. Dotted lines represent the genome-wide significance threshold of *P* < 5.0 × 10^−8^.

We enrolled 2,393 unrelated patients with COVID-19 who required hospitalization between April 2020 and January 2021 (during the first, second and third waves of the pandemic in Japan) to the GWAS, from more than 100 hospitals participating in the JCTF. The COVID-19 diagnoses were confirmed by physicians at each affiliated hospital on the basis of clinical manifestations and a positive PCR test result. As controls, we enrolled 3,289 unrelated subjects ahead of the COVID-19 pandemic, representative of the general Japanese population. All of the participants were confirmed to be of East Asian origin by principal component analysis (Extended Data Fig. 2a,b).

Of the 2,393 patients with COVID-19, 990 had severe infection as defined by the need for oxygen support, artificial respiration and/or intensive care, whereas 1,391 patients had non-severe disease. Severity information was not available for the remaining 12 individuals. As reported previously^8,10^, those with severe COVID-19 were older (65.3 ± 13.9 years (mean ± s.d.)) and included a higher proportion of males (73.9%) compared with non-severe cases (49.3 ± 19.2 years of age and 57.2% male).

To replicate these results, we enrolled 1,243 further patients with severe COVID-19 collected between February 2021 and September 2021 (the fourth and fifth waves of the pandemic in Japan) and 3,769 controls. Detailed characteristics of the participants are provided in Supplementary Table 2.

## COVID-19 GWAS in the Japanese population

**Fig. 1:**
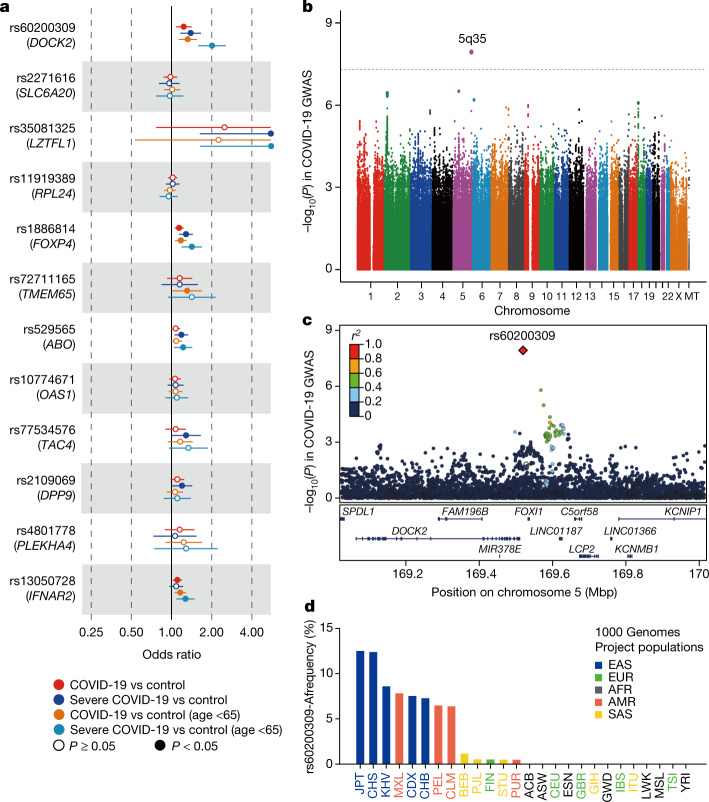
GWAS in a Japanese population stratified by COVID-19 severity and age. **a**, Forest plots of the risk of COVID-19-associated variants in a Japanese population. Error bars indicate the 95% confidence interval. **b**, Manhattan plot of the GWAS on severe COVID-19 in young patients (those less than 65 years of age) (440 cases and 2,377 controls). Uncorrected *P* values from the GWAS analysis are shown. The dotted line represents the genome-wide significance threshold of *P* < 5.0 × 10^−8^. Manhattan and quantile–quantile plots of all GWAS results are presented in Extended Data Fig. 2. MT, mitochondrial. **c**, Regional association plot at the *DOCK2* locus. Dots represent SNPs coloured according to linkage disequilibrium (*r*^*2*^) with the lead SNP of rs60200309. *FAM196B* is also known as *INSYN2B*. **d**, Allele frequency spectra of the rs60200309-A allele in the 1000 Genomes Project Phase3v5 database.

The GWAS including all COVID-19 cases yielded no signals satisfying a genome-wide significance threshold (*P* < 5.0 × 10^−8^; Extended Data Fig. 2c). Cross-population comparisons confirmed the risks at multiple COVID-19-associated variants identified in the previous studies^1,3,5^. Seven out of the eleven reported positive associations were replicated in our Japanese cohort with *P* < 0.05, including those at *LZTFL1*, *FOXP4*, *TMEM65*, *ABO*, *TAC4*, *DPP9* and *IFNAR2* (Fig. 1a and Supplementary Table 3), where the highest odds ratios were observed in comparisons for severe and young (less than 65 years of age) COVID-19 cases in 6 out of the 7 loci. The most significant replication was observed at *FOXP4*, as expected from its higher allele frequency in East Asian people than in Europeans^3^ (odds ratio = 1.29, 95% confidence interval 1.13–1.46, *P* = 9.1 × 10^−5^ for severe COVID-19). By contrast, the risk allele at *LZTFL1* (rs35081325), which showed the strongest association in Europeans, was rare in Japanese patients. Despite its low frequency (0.0013 in controls), we nominally replicated the association with the highest risk in the young patients with severe COVID-19 (odds ratio = 11.8, 95% confidence interval = 1.64–85.5, *P* = 0.014).

**Extended Data Fig. 3:**
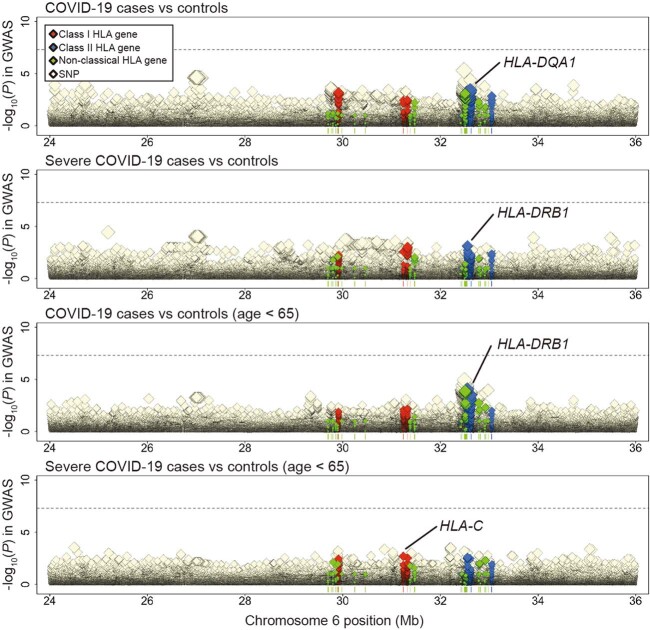
Regional association plots of the HLA imputation analysis. Regional association plots of the HLA imputation analysis results. Dots represent SNPs and HLA variants with colors according to the legend. Uncorrected *P* values from HLA imputation analysis are shown. Dotted lines represent the genome-wide significance threshold of *P* < 5.0 × 10^−8^. HLA genes with the most significant associations in each of the case-control phenotypes are indicated.

**Extended Data Fig. 4:**
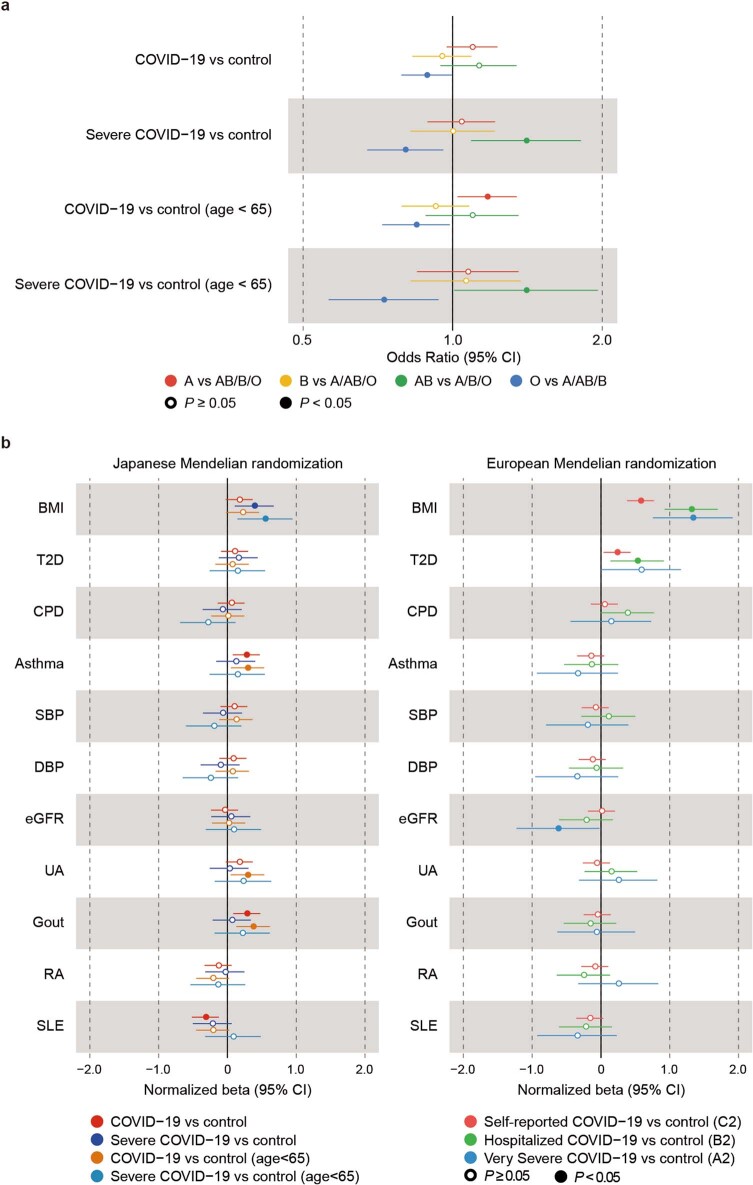
ABO blood type associations with COVID-19 in Japanese and cross-population Mendelian randomization analysis of the COVID-19 GWAS. (**a**) Odds ratios of the ABO blood types in the Japanese population are indicated. Dots represent the odds ratios and bars represent the 95 % confidence intervals. *P* values are uncorrected. Detailed results are presented in Supplementary Table 5. (**b**) Forest plots of the Mendelian randomization (MR) analysis results of causal inference on the COVID-19 GWAS in Japanese (left panel) and Europeans (right panel). Since effect sizes (= beta) of MR are not scalable among phenotypes and populations, normalized beta is indicated. For each phenotype and population, the standard error for the COVID-19 GWAS with the largest sample size (i.e., “COVID-19 vs control” for Japanese and “Self-reported COVID-19 vs control (C2)” for Europeans) was set to be 0.1. Dots represent the effect size normalized beta estimates and bars represent the 95 % confidence intervals. *P* values are uncorrected. The abbreviations of the exposure phenotypes and the detailed MR results are given in Supplementary Table 6 and Supplementary Table 7. BMI; body mass index, T2D; type 2 diabetes, CPD; cigarettes per day, CAD; cardiovascular disease, SBP; systolic blood pressure, DBP; diastolic blood pressure, eGFR; estimated glomerular filtration rate, UA; serum uric acids, RA; rheumatoid arthritis, SLE; systemic lupus erythematosus.

We evaluated the effects of human leukocyte antigen (HLA) variants on COVID-19 risk^11,12^ by in silico HLA imputation analysis^13,14^. We did not observe association signals satisfying the HLA-wide significance threshold (*P* < 0.05 over 2,482 variants, 2.0 × 10^−5^; Extended Data Fig. 3 and Supplementary Table 4). Among the four major ABO blood types^15^, the O blood type was associated with a protective effect (*P* < 0.05), most evidently in young patients with severe COVID-19^1^ (odds ratio = 0.73, 95% confidence interval 0.56–0.93, *P* = 0.014; Extended Data Fig. 4a and Supplementary Table 5). We found an increased risk associated with the AB blood type, especially in severe cases of COVID-19 (odds ratio = 1.41, 95% confidence interval 1.10–1.81, *P* = 0.0065 for all ages). The Japanese population has the highest frequency of the AB blood type^16^ (9.5% in our study), which may have provided the power to detect its risk.

## Cross-population Mendelian randomization

Next, to identify medical conditions that may affect COVID-19 susceptibility, we applied cross-population two-sample Mendelian randomization analysis^17^ (Supplementary Table 6). We inferred a causal role for obesity in severe COVID-19 in the Japanese cohort (*P* < 0.0074; Extended Data Fig. 4b and Supplementary Table 7). We also inferred causal roles for asthma, uric acids and gout, whereas systemic lupus erythematosus showed a protective effect (*P* < 0.05). Hyperuricemia is a risk factor for severe COVID-19 in the Japanese population^10^, consistent with our findings from Mendelian randomization. In Europeans, we observed significant causal inferences for obesity^18^ (*P* < 6.2 × 10^−6^), with doubled effect sizes in hospitalized patients and those with severe COVID-19 when compared with self-reported COVID-19. Our analysis provided additional evidence of obesity as a risk factor^8,9^.

## A population-specific risk allele on *DOCK2*

Given the observation that many COVID-19 risk variants confer larger effects in severe disease and young patients^1,3,5,19^, we stratified the subjects according to age and disease severity, analysing those with severe COVID-19 (*n* = 990), young patients^9^ (*n* = 1,484) and young patients with severe COVID-19 (*n* = 440).

By comparing young patients with severe COVID-19 and controls, we identified a genetic locus on 5q35 that satisfied genome-wide significance (*P* = 1.2 × 10^−8^ at rs60200309; Fig. 1b). The A allele of the lead SNP (rs60200309), located at an intergenic region downstream of *DOCK2*, was associated with an increased risk of severe COVID-19 (odds ratio = 2.01, 95% confidence interval 1.58–2.55, *P* = 1.2 × 10^−8^; Fig. 1c and Table 1). The rs60200309-A allele was also associated with an increased risk of COVID-19 in other comparisons, including all COVID-19 cases and controls (odds ratio = 1.24; Supplementary Table 8), and within-case severity analysis (that is, severe versus non-severe cases; odds ratio = 1.27 for all ages and odds ratio = 1.90 for ages < 65 years).

**Table 1:** Association of the *DOCK2* variant with COVID-19 risk in the Japanese population

rsID Chromosome position Cytoband allele Gene	Case collection period	Age	Phenotype	No. of subjects		Risk allele frequency (A)		Odds ratio (95% confidence interval)	*P* value
				Cases	Controls	Cases	Controls		
rs60200309 5:169519612 5q35 G/A *DOCK2*	GWAS (April 2020 to January 2021)	All ages	COVID-19 vs control	2,393	3,289	0.12	0.10	1.24 (1.09–1.41)	0.0011
			Severe COVID-19 vs control	990	3,289	0.13	0.10	1.39 (1.16–1.66)	3.1 × 10^−4^
		<65 years	COVID-19 vs control	1,484	2,377	0.12	0.10	1.32 (1.13–1.55)	5.1 × 10^−4^
			Severe COVID-19 vs control	440	2,377	0.16	0.10	2.01 (1.58–2.55)	1.2 × 10^−8^
	Replication (February 2021 to September 2021)	All ages	Severe COVID-19 vs control	1,243	3,769	0.11	0.11	1.00 (0.85–1.19)	0.96
		<65 years		833	1,242	0.12	0.10	1.28 (1.02–1.61)	0.033

Uncorrected *P* values are shown.

**Extended Data Fig. 5:**
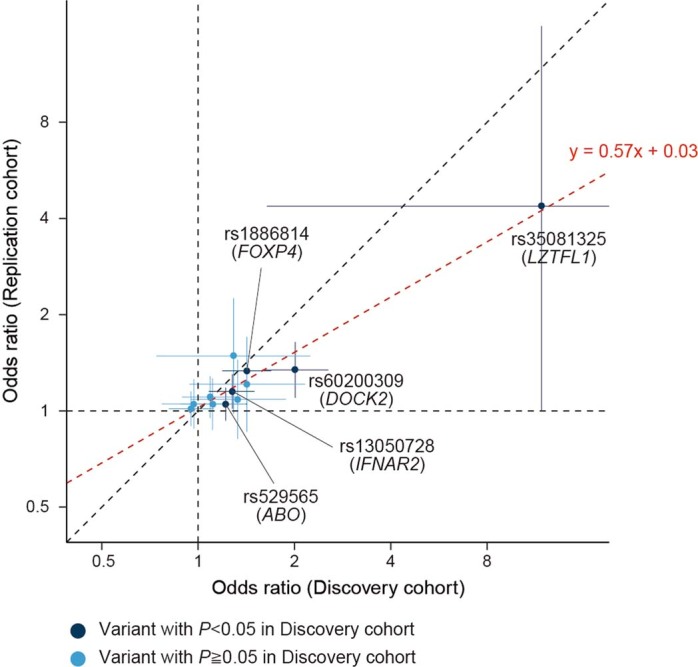
Effect size comparisons of the COVID-19 risk loci between the discovery GWAS and the replication study. Co-plots of the odds ratios and 95% confidence intervals between the discovery GWAS cohort and replication cohort. To focus on the differences in the cases collected in different pandemic waves (initial waves for GWAS and latter waves for the replication), same controls as GWAS were currently used for the cases in the replication. A regression coefficient was estimated based on logarithm of odds ratios. Dots represent the odds ratios and bars represent the 95 % confidence intervals.

We then conducted a replication study using an additional 1,243 patients with severe COVID-19, recruited during the fourth and fifth waves of the pandemic, as well as 3,769 controls. We replicated an age-specific nominal risk in the young patients with COVID-19 (*n* = 833; odds ratio = 1.28, 95% confidence interval 1.02–1.61, *P* = 0.033; Table 1) compared with all ages (odds ratio = 1.00, 95% confidence interval 0.85–1.19, *P* = 0.96), whereas the effect size was smaller than that observed in the GWAS during the first three pandemic waves. A decreased severity risk was observed for other risk loci in this later study (for example, odds ratios of 11.8 during the first three waves and 4.4 during the fourth and fifth waves at *LZTFL1*; regression coefficient = 0.57; Extended Data Fig. 5). This suggests that longitudinal shifts of confounding factors with the pandemic waves—such as the introduction of therapeutic strategies, a high prevalence of vaccination, changes in hospitalization policy and the evolution of virus strains—may have mitigated the host genetic burdens defined during the initial pandemic waves; further evaluations of this effect may be warranted.

We also examined the COVID-19 risk profile of the *DOCK2* variant on different ancestral backgrounds^20,21^ (3,138 hospitalized patients with COVID-19 versus 891,375 controls from the pan-ancestry meta-analysis). We observed the same directional effect, with a marginal association signal (odds ratio = 1.73, 95% confidence interval 0.95–3.15, *P* = 0.072, control minor allele frequency (MAF) = 0.0008; Supplementary Table 9).

The *DOCK2* variant was prevalent in East Asian people (0.097)—with the highest frequency (0.125) in Japanese individuals—and, to a lesser extent, in Native Americans (0.049), but was very rare in other groups (<0.005; Fig. 1d). Natural selection screening in Japanese participants^22^ suggested marginal positive selection of the variant (*P* for singleton density score = 0.051). Population-specific features of the *DOCK2* variant provide a rationale for COVID-19 host genetic research in non-European populations.

## *DOCK2* downregulation in severe COVID-19

**Fig. 2:**
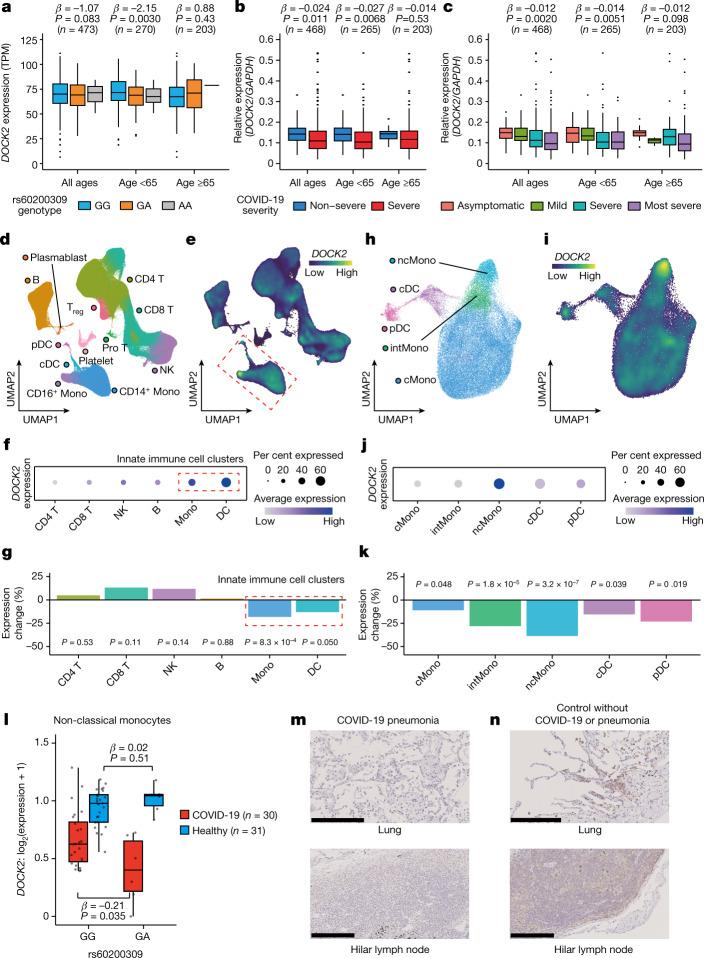
Cell-type- and tissue-specific expression of *DOCK2* and its downregulation in severe COVID-19. **a**, eQTL effect of the COVID-19 risk variant (rs60200309) on *DOCK2* expression levels using bulk RNA-seq of peripheral blood. The risk allele (rs60200309-A) decreases *DOCK2* levels in patients with COVID-19 aged below 65 years. TPM, transcripts per kilobase million. **b**,**c**, Differential expression analysis of *DOCK2* with varying COVID-19 severity. *DOCK2* expression levels were quantified by qPCR and normalized to *GAPDH* expression. **b**, Comparison between severe and non-severe COVID-19 cases. **c**, Comparison between most severe, severe, mild and asymptomatic cases of COVID-19. **d**–**k**, scRNA-seq in PBMCs from individuals with severe COVID-19 (*n* = 30) and healthy controls (*n* = 31). **d**, Uniform manifold approximation and projection (UMAP) visualization of all 394,526 cells. **e**, Projection of *DOCK2* gene expression. Innate immune cell clusters are outlined with a red dashed line. **f**, Percentage of *DOCK2*-expressing cells and *DOCK2* expression levels. **g**, Expression change with severe COVID-19 in six major cell types. **h**, Visualization and annotation of the innate immune cell clusters. **i**–**k**, *DOCK2* expression and expression changes with severe COVID-19 in the innate immune cell clusters. **i**, Projection of *DOCK2* gene expression. **j**, Percentage of *DOCK2*-expressing cells and *DOCK2* expression levels. **k**, Expression change with severe COVID-19 in five cell types. **l**, COVID-19 context-specific decreasing eQTL effect of the *DOCK2* risk variant in non-classical monocytes. **m**,**n**, Immunohistochemical analysis of DOCK2. Lung and hilar lymph nodes were obtained from patients with COVID-19 pneumonia (**m**) or controls without COVID-19 or pneumonia (**n**), and stained with anti-DOCK2 polyclonal antibody. Results for all samples are shown in Extended Data Fig. 9. Scale bars, 0.25 mm. In **a**–**c**,**l**, boxes denote the interquartile range (IQR) and the median is shown as horizontal bars; whiskers extend to 1.5 times the IQR; outliers are shown as individual points in **a**–**c** and all samples are shown as individual points in **l**. Uncorrected *P* values are shown in (**a**–**c**,**g**,**k**,**l**). cDC, conventional dendritic cells; cMono, classical monocytes; intMono, intermediate monocytes; Mono, monocytes; ncMono, non-classical monocytes; NK, natural killer cells; Pro T, proliferative T cells; T_reg_, T regulatory cells.

**Extended Data Fig. 6:**
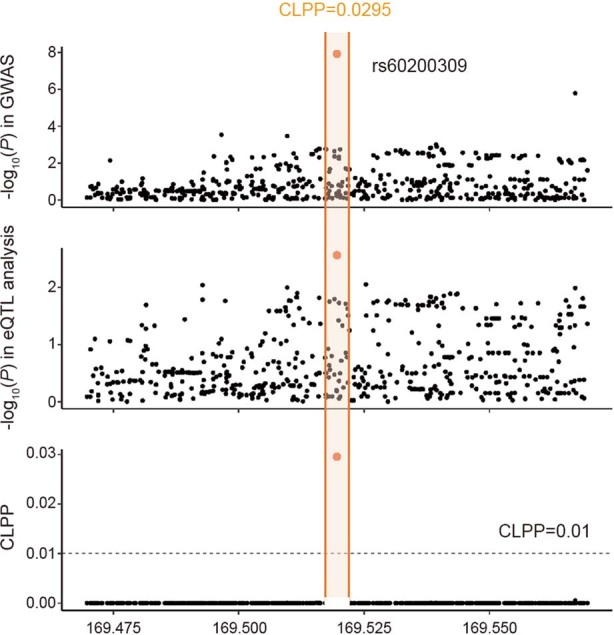
Colocalization analysis of the GWAS and eQTL signals at the *DOCK2* locus. Regional colocalization plots of the GWAS signals (severe and younger COVID-19 cases vs controls) and the eQTL signals on *DOCK2* expression in the COVID-19 patients at the *DOCK2* locus. CLPP; colocalization posterior probability. The eQTL effects of the variants around *DOCK2* region are given in Supplementary Table 10.

To functionally annotate the *DOCK2* risk variant, we examined the expression quantitative trait loci (eQTL) effect by conducting peripheral blood RNA-sequencing (RNA-seq) analysis of data from patients with COVID-19 collected by the JCTF (*n* = 473). The risk allele at *DOCK2* (rs60200309-A) was not associated with a significant eQTL effect for all patients (β = −1.07, *P* = 0.083; Fig. 2a), but was associated with decreased expression of *DOCK2* in the patients below 65 years of age (*n* = 270; β = −2.15, *P* = 0.0030). This allele did not exhibit a significant eQTL effect on other surrounding genes (±500 kb window, *P* > 0.070). We observed colocalization between the GWAS and the *DOCK2* eQTL signals^23^ (colocalization posterior probability > 0.01; Extended Data Fig. 6 and Supplementary Table 10).

We analysed differential expression of *DOCK2* in patients with severe and non-severe COVID-19 (*n* = 468) using real-time quantitative PCR (qPCR). *DOCK2* expression was reduced in the patients with severe COVID-19 (*P* = 0.011; Fig. 2b). Suppression of *DOCK2* was more marked in young patients (*P* = 0.0068). When the patients were further stratified into asymptomatic, mild, severe and most severe cases, we observed a negative correlation between *DOCK2* expression level and disease severity (Fig. 2c). Together, these results indicate that *DOCK2* expression is downregulated in peripheral blood cells of patients with severe COVID-19, especially in young patients, and that the risk variant may contribute to severe COVID-19 by suppressing expression of *DOCK2*.

**Extended Data Fig. 7:**
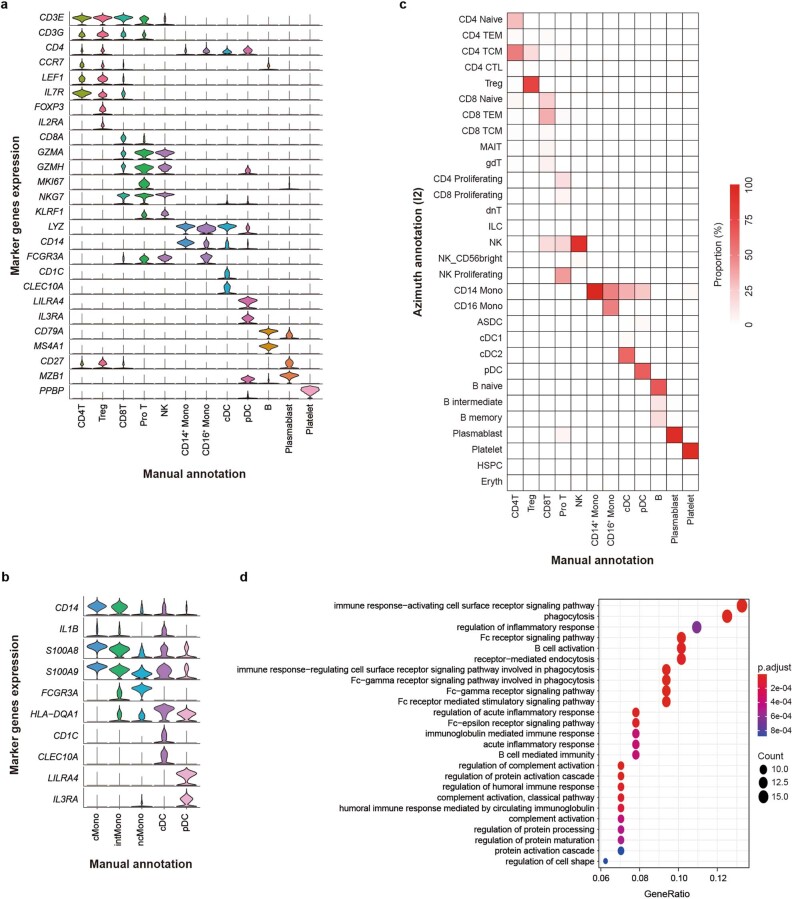
Cell type definition and gene ontology enrichment analysis of *DOCK2* co-expression gene module in the PBMC single cell analysis. (**a**) Violin plots showing the expression distribution of selected canonical cell markers in the 12 clusters of PBMC. The rows represent selected marker genes and the columns represent clusters with the same color as in Fig. 2d. (**b**) Violin plots showing the expression distribution of selected canonical cell markers in the 5 clusters of innate immune cell clusters, shown in the same color as in Fig. 2h. (**c**) Tile plot showing percentage concordance between the manually annotated 12 clusters and Azimuth annotation. (**d**) The top 25 enriched biological processes by gene ontology (GO) analysis of *DOCK2* co-expression gene module identified by weighted gene co-expression network analysis (WGCNA) in the non-classical monocytes of COVID-19 patients, where *DOCK2* showed the highest cell type-specific expression profile. The color of the dots represents the adjusted *P* values.

DOCK2 is a RAC activator that is involved in chemokine signalling, production of type I interferon (IFN) and lymphocyte migration^24,25^. Elucidation of immune cell-type-specific expression profiles was necessary to disentangle the roles of DOCK2 in the biology of COVID-19. We therefore conducted single-cell RNA-seq (scRNA-seq) of peripheral blood mononuclear cells (PBMC) obtained from 30 patients with severe COVID-19 and 31 healthy controls. We obtained 394,526 high-quality single cells and annotated 12 clusters (Fig. 2d and Extended Data Fig. 7). *DOCK2* expression was highest in CD16^+^ monocytes (Fig. 2e). The proportion of cells expressing *DOCK2* was higher in innate immune cell clusters (monocytes and dendritic cells) (43.8%) than in other clusters (25.6%; Fig. 2f). Differential expression analysis also demonstrated suppression of *DOCK2* expression in cases of severe COVID-19 in the immune cell clusters (fold change (FC) = 0.82, *P* = 8.3 × 10^−4^ for monocytes; FC = 0.87, *P* = 0.050 for dendritic cells; Fig. 2g).

To determine immune cell-type specificity, we performed clustering and annotation by extracting 63,544 cells belonging to the innate immune cell clusters (Fig. 2h and Extended Data Fig. 7). Among the classified cell types—classical (CD14^++^CD16^–^), intermediate (CD14^++^CD16^+^) and non-classical (CD14^+^CD16^++^) monocytes, conventional dendritic cells and plasmacytoid dendritic cells (pDCs)—*DOCK2* expression was highest in the non-classical monocytes, which have been implicated in the pathophysiology of COVID-19 (refs. ^26,27^) (Fig. 2h–j). Differential expression analysis showed that *DOCK2* was most potently downregulated in non-classical monocytes (FC = 0.61, *P* = 3.2 × 10^−7^; Fig. 2k). The *DOCK2* co-expression gene module^28^ in the non-classical monocytes of the COVID-19 patients exhibited enrichment in pathways such as immune response signalling pathways and phagocytosis (Extended Data Fig. 7). To further support the functional consequences of the *DOCK2* risk variant, we assessed its single-cell eQTL effects. We found a COVID-19 context-specific decreasing dosage effect of the risk variant on *DOCK2* expression in non-classical monocytes (β = −0.21, *P* = 0.035 for COVID-19 and β = 0.02, *P* = 0.51 for controls; Fig. 2l).

**Extended Data Fig. 8:**
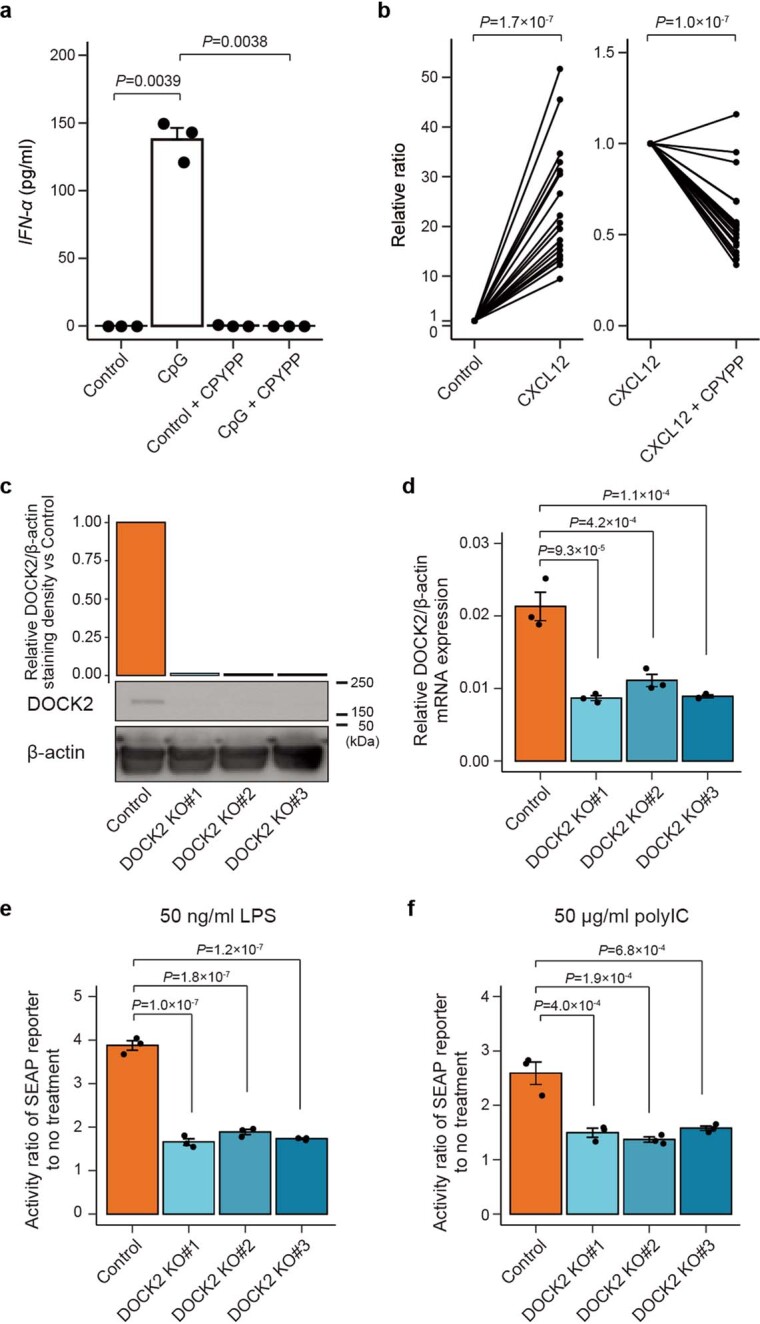
Biological impacts of *DOCK2* downregulation in primary cells and *DOCK2* knockdown and Interferon-α production assay in THP-1 Blue ISG cells. (**a**) The impact of *DOCK2* downregulation on interferon-α (*IFN-α*) production ability in pDC. Sorted pDC were stimulated with CpG and/or CPYPP. Data shows means ± s.e.m. (*n* = 3 per group). Differences of *IFN-α* production ability between the groups were evaluated using two-sided paired *t*-test. (**b**) The impact of *DOCK2* downregulation on chemotaxis in CD3^+^ T cells. CD3^+^ T cells were stimulated with CXCL12 or CXCL12 + CPYPP (*n* = 19 per group). Differences of chemotaxis between the groups were evaluated using two-sided paired *t*-test. (**c**, **d**) Knockdown of *DOCK2* by CRISPR system was confirmed by western blotting (**c**) and qRT-PCR. (**d**) Semi-quantitative staining density measure was determined using ImageJ (NIH). Data shows means ± s.e.m. (*n* = 3 per group). Data are compared to control group. *P* values were determined with One-way ANOVA followed by Dunnett’s post hoc test. (**e**, **f**) Activity ratio of SEAP reporter to no treatment group. Reporter was activated by 50 ng/ml LPS (**e**) or 50 *μ*g/ml polyIC (**f**). Data shows means ± s.e.m. (*n* = 3 per group). Data are compared to control group. *P* values were determined with One-way ANOVA followed by Dunnett’s post hoc test.

Next, we evaluated the biological effects of *DOCK2* downregulation. In assays with primary cells, *DOCK2* inhibition by CPYPP, an inhibitor of the DOCK2–RAC1 interaction^29^, resulted in reduced production of IFNα by pDCs under CpG stimulation (FC = 5.5 × 10^−5^, *P* = 0.0038, *n* = 3 per group; Extended Data Fig. 8a). pDCs are another key innate immune cell type involved in COVID-19 pathogenicity^30^, and *DOCK2* expression was downregulated in pDCs from patients with COVID-19 (FC = 0.79, *P* = 0.019; Fig. 2k). CPYPP blocked chemotaxis of CD3^+^ T cells under CXCL12 stimulation (FC = 0.57, *P* = 1.0 × 10^−7^, *n* = 19 per group; Extended Data Fig. 8b). The *DOCK2* risk variant had no significant effect on IFNα production in pDCs or chemotaxis of CD3^+^ T cells in primary cell assays (Supplementary Fig. 1). In THP1 Blue ISG cells, *DOCK2* knockdown caused a marked decrease in transcriptional activation of IFN-stimulated genes, an indicator of type I IFN activity (Extended Data Fig. 8c–f and Supplementary Fig. 2). These results highlight the immunological roles of DOCK2 in complications of COVID-19 such as type I IFN immunity and chemotaxis dysregulation, as exemplified by patients with congenital impairment in type I IFN immunity^31^.

**Extended Data Fig. 9:**
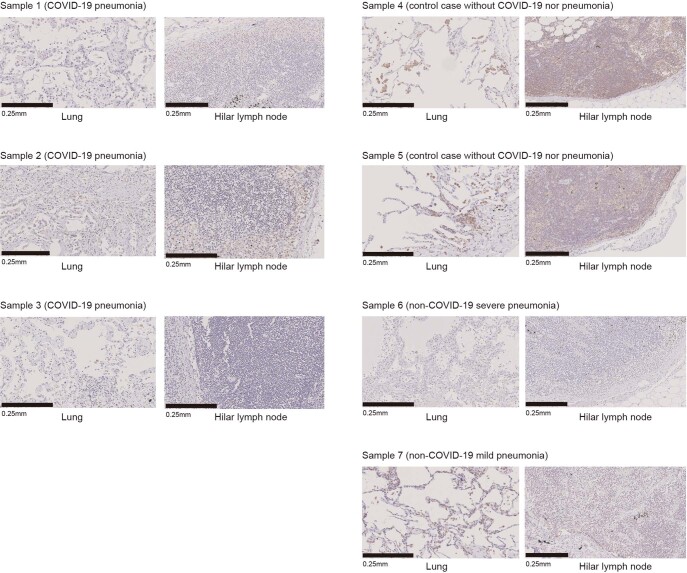
Immunohistochemical analysis for DOCK2. Lung and hilar lymph nodes were obtained from autopsied cadaver (Sample 1–3, 6, 7) or surgical specimen (Sample 4, 5), and stained by anti-DOCK2 polyclonal antibody. Sample 1–3; COVID-19 pneumonia. Sample 4-5; control. Sample 6; non-COVID-19 severe pneumonia. Sample 7; non-COVID-19 mild pneumonia.

To confirm the involvement of DOCK2 in COVID-19 pneumonia, we performed immunohistochemical analysis on postmortem samples from people who died from COVID-19 (Extended Data Fig. 9). We examined three cases of COVID-19 pneumonia and observed decreased expression of DOCK2 in lymphocytes and macrophages located in the lung and in hilar lymph nodes (Fig. 2m). There was no such decrease in two control samples without COVID-19 or pneumonia (Fig. 2n). DOCK2 has been reported to be suppressed in bronchoalveolar lavage fluid cells of patients with COVID-19 (ref. ^32^), consistent with our findings. We observed a loss of *DOCK2* expression in lymphocytes in a case of non-COVID-19 severe pneumonia, whereas there was a slight decrease of *DOCK2* expression in a sample from a case of non-COVID-19 mild pneumonia. Thus, DOCK2 expression is suppressed during severe pneumonia caused by COVID-19. These observations reveal a link between cell-type- and tissue-specific downregulation of DOCK2, indicating a potential value for DOCK2 as a biomarker of severe COVID-19.

## DOCK2 inhibition in a Syrian hamster model

**Fig. 3:**
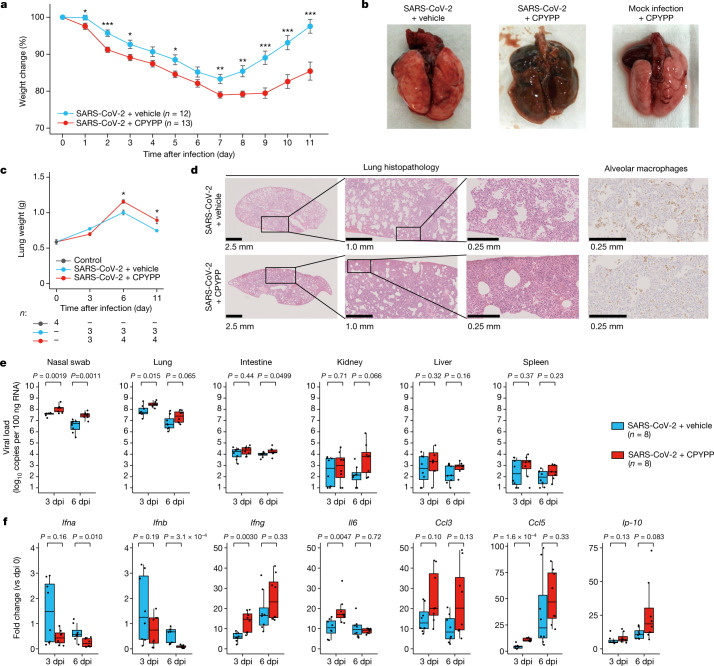
In vivo suppression of *DOCK2* in a Syrian hamster model of SARS-CoV-2 infection. **a**, Changes in body weight of hamsters infected with SARS-CoV-2. **b**, Representative images of lungs collected after euthanizing the hamsters at 11 dpi. **c**, Lung weight changes after infection. The number of samples (*n*) is indicated. **d**, Representative lung histopathology and immunohistochemistry of the infected hamsters at 6 dpi. Outlined areas are expanded to the right of each image. Right, lung tissue was stained with the anti-CD68 mouse monoclonal antibody to highlight alveolar macrophages. **e**, SARS-CoV-2 viral loads in the organs of the infected hamsters at 3 and 6 dpi. **f**, Lung cytokine expression assays of the infected animals. *Ip-10* is also known as *CXCL10*. In (**a**) and (**c**), the error bars represent standard error of the mean, and *P* values were determined with two-sided Welch’s *t*-test; *P < 0.05; **P < 0.01; ***P < 0.001. In (**e**) and (**f**), boxes denote the IQR, and the median is shown as horizontal bars. Whiskers extend to 1.5 times the IQR, and all animals are shown as individual points. *P* values were determined with two-sided Wilcoxon rank sum test.

**Extended Data Fig. 10:**
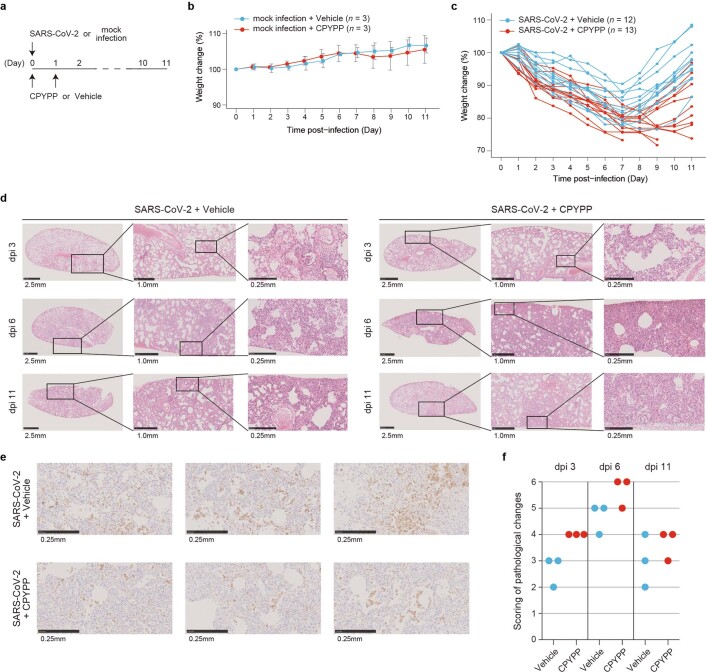
*In vivo* suppression of DOCK2 in a Syrian hamster model with SARS-CoV-2 infection. (**a**) Schematic timeline of the experimental procedure. (**b**) Changes in weight of uninfected animals. The error bars represent standard error of the mean. (**c**) Changes in weight of each of the infected animals, corresponding to Fig. 3a. Three CPYPP-administrated animals reaching humane endpoint were euthanized at dpi 7 and 9, lowering survival rate to 77% (=10/13), while survival of vehicle-administrated animals was 100% (=12/12). The animals were administered with CPYPP (red), or vehicle (blue). (**d**) Histopathological examination of the lungs of infected hamsters. Syrian hamsters were inoculated with SARS-CoV-2 with CPYPP or Vehicle. Syrian hamsters infected with CPYPP or Vehicle were euthanized on dpi 3, 6, and 11 for pathological examinations (*n* = 3). Shown are pathological findings in the lungs of hamsters infected with the virus on dpi 3, 6, and 11 (hematoxylin and eosin staining). Middle and Right show enlarged views of the area circled in black in Left. (Scale bars, 2.5 mm [Left], 1.0 mm [Middle], and 0.25 mm [Right].) (**e**) Immunohistochemistry for alveolar macrophages. Shown are immunohistochemical findings in the lungs of hamsters infected with the virus on dpi 6 (*n* = 3 per group). Lung tissue was stained with the anti-CD68 mouse monoclonal antibody. (Scale bars, 0.25 mm.) (**f**) Pathological severity scores in infected hamsters. To evaluate comprehensive histological changes, lung tissue sections were scored based on (**d**) pathological changes. Scores were determined based on the percentage of inflammation area of the maximum cut surface collected from each animal in each group by using the following scoring system: 0, no pathological change; 1, affected area (≤10%); 2, affected area (<50%, > 10%); 3, affected area (<90%, ≥50%); 4, (≥90%) an additional point was added when pulmonary edema and/or alveolar hemorrhage was observed. The total score is shown for individual animals. Blue dot and red dot indicate +Vehicle and +CPYPP, respectively.

To decipher in vivo pathogenesis of *DOCK2* in COVID-19, we investigated the effects of DOCK2 suppression following SARS-CoV-2 infection in a Syrian hamster model^33,34^ (Extended Data Fig. 10a). Administration of the DOCK2 inhibitor CPYPP or vehicle (as a negative control) to mock-infected animals did not induce weight loss (Extended Data Fig. 10b). However, hamsters infected with SARS-CoV-2 and treated with vehicle (*n* = 12) decreased to 83.3% of the starting body weight by 7 days post-infection (dpi), but recovered to 97.6% of the starting weight at 11 dpi. By contrast, hamsters infected with SARS-CoV-2 and treated with CPYPP (*n* = 13) decreased to 79.0% of the starting body weight by 7 dpi, and recovered to 85.4% of the initial weight at 11 dpi (Fig. 3a and Extended Data Fig. 10c). Advanced pulmonary oedema was observed in the lung of the hamsters infected with SARS-CoV-2 and treated with CPYPP at 11 dpi (Fig. 3b). The largest lung weight (Fig. 3c) and the highest histopathological scoring changes of lung^34^ (Fig. 3d and Extended Data Fig. 10d–f) were observed at 6 dpi. Lung immunohistochemistry showed that the migration of CD68 macrophages around alveolar cells was impaired in the hamsters infected with SARS-CoV-2 and treated with CPYPP (Fig. 3d and Extended Data Fig. 10e). Conversely, there was mild or no lung damage in infected hamsters treated with vehicle or uninfected hamsters treated with CPYPP (Fig. 3b–d and Extended Data Fig. 10d–f).

Focusing on the deteriorating stages of SARS-CoV-2-induced pneumonia (3 and 6 dpi), we assayed SARS-CoV-2 viral loads in various organs. We observed increased viral loads in nasal swab at 3 and 6 dpi, in lung at 3 dpi and in intestine at 6 dpi (*P* < 0.05; Fig. 3e) of the CPYPP-treated hamsters. Lung cytokine expression profile assays revealed that expression of type I IFN (encoded by *Ifna* and *Ifnb*) decreased at 6 dpi and expression of type II IFN (encoded by *Ifng*) increased at 3 dpi (Fig. 3f) following CPYPP administration. We also observed that CPYPP administration induced increased expression of inflammatory cytokine (*Il6*) and chemokine (*Ccl5*) genes at 3 dpi. The roles of the IFN response in the pathogenicity of COVID-19 have been controversial^31,35,36^. Our observational and interventional findings on *DOCK2* downregulation show that in COVID-19 pneumonia pathophysiology, impaired macrophage recruitment at the site of infection and dysregulated IFN responses result in impaired virus elimination and prolonged lung inflammation.

## Discussion

Here we reported on a GWAS of COVID-19 in a Japanese cohort, one of the first large-scale COVID-19 genetic studies in a non-European population. We confirmed the presence of multiple genetic variants associated with COVID-19 risk shared across different populations, identified a population-specific risk variant at *DOCK2*, particularly in young patients with severe COVID-19 collected during the early waves of the pandemic. Cross-population Mendelian randomization analysis disclosed causal effects of a number of complex human traits, such as obesity, on COVID-19. Our results highlight the role of population-specific risk alleles on different host genetic backgrounds, underscoring the need for studies of COVID-19 host genetics in non-European populations. Of note, autosomal recessive *DOCK2* deficiency is a Mendelian disorder associated with combined immunodeficiency and severe invasive pneumonia^37^ (Online Mendelian Inheritance in Man (OMIM) entry 616433). Our results provide a genetic and clinical link between a Mendelian disorder and pneumonia associated with COVID-19. In the replication study using samples collected during later waves of the COVID-19 pandemic, we observed significant increases in the risk of severe COVID-19 associated with the risk variants identified in the studies based on the initial waves—including variants in *DOCK2* and *LZTFL1*—but with smaller effect sizes. How the host genetics interact longitudinally with confounding factors and affect the spectrum of COVID-19 phenotypes through the pandemic waves remains unknown. Large-scale COVID-19 host genetics studies with diverse genetic backgrounds based on samples from different time points during the pandemic are required, and will contribute towards planning a global health strategy for the pandemic.

Our follow-up analyses of GWAS showed that DOCK2-mediated signalling has a key role in the response to SARS-CoV-2 infection, suggesting that the hypomorphic *DOCK2* allele is involved in exacerbation of COVID-19 pathology, and that *DOCK2* could serve as a potential clinical biomarker to predict severe COVID-19. Bulk and single-cell transcriptome analysis of peripheral blood cells identified cell-type-specific downregulation of *DOCK2* modulated by a COVID-19-specific eQTL effect of the *DOCK2* risk variant in patients with severe COVID-19, which was most evident in innate immune cells including non-classical monocytes and pDCs. Nevertheless, our evidence does not necessarily imply a direct causal link between the COVID-19-specific eQTL and COVID-19 severity. The risk variant could potentially induce *DOCK2* downregulation in early phase of infection. Immunohistochemical analysis showed reduced DOCK2 expression in the lung of patients with COVID-19 pneumonia. In vivo inhibition of DOCK2 activity following SARS-CoV-2 infection using CPYPP in the Syrian hamster model resulted in severe COVID-19 pneumonia, highlighted by impaired migration of macrophages and dysregulation of the IFN response. We note the possibility that CPYPP is not specific to DOCK2 and also inhibits other DOCK family proteins. Assays with increased *DOCK2* expression would provide further evidence of its role in COVID-19 pathophysiology. Given its critical roles in immune regulation^25^, upregulation of DOCK2 could be a potential therapeutic strategy against COVID-19. Our results motivate further studies linking DOCK2 to molecular and clinical phenotypes of COVID-19 in the effort to overcome the pandemic.

## Methods

### Study participants

All the cases affected with COVID-19 were recruited through the JCTF. We enrolled hospitalized patients diagnosed as COVID-19 by physicians using the clinical manifestation and PCR test results who were recruited at any of the more than 100 affiliated hospitals between April 2020 and January 2021 (for the GWAS) or between February 2021 and September 2021 (for the replication; Supplementary Tables 1 and 2). Patients requiring oxygen support, artificial respiration and/or intensive care unit hospitalization were defined as having ‘severe COVID-19’, whereas others were defined as having ‘non-severe COVID-19’. Details of the clinical manifestation including cardiovascular and respiratory comorbidities are provided in Supplementary Table 2. The threshold of 65 years of age was selected according to the clinical management guide in Japan^9^. Control subjects were collected from the general Japanese population at Osaka University and affiliated institutes (for the GWAS and replication) or by the Biobank Japan Project^38^ (for the replication). Individuals determined to be of non-Japanese origin either by self-reporting or by principal component analysis were excluded as described elsewhere^39^ (Extended Data Fig. 2a). All the participants provided written informed consent as approved by the ethical committees of the affiliated institutes (Keio IRB approval 20200061, Osaka University IRB approval 734-14, University of Tsukuba IRB approval H29-294).

### GWAS genotyping and QC

We performed GWAS genotyping of the 2,520 COVID-19 cases and 3,341 controls using Infinium Asian Screening Array (Illumina). We applied stringent quality control (QC) filters to the samples (sample call rate < 0.97, excess heterozygosity of genotypes >mean + 3 × s.d., related samples with PI_HAT > 0.175, or outlier samples from East Asian clusters in principal component analysis with 1000 Genomes Project samples), and variants (variant call rate < 0.99, significant call rate differences between cases and controls with *P* < 5.0 × 10^−8^, deviation from Hardy–Weinberg equilibrium with *P* < 1.0 × 10^−6^, or minor allele count <5). Details of the QC for the mitochondrial variants are described elsewhere^40^. After QC, we obtained genotype data of 489,539, 15,161 and 217 autosomal, X-chromosomal and mitochondrial variants, respectively, for 2,393 COVID-19 cases and 3,289 controls.

### Genome-wide genotype imputation

We used SHAPEIT4 software (version 4.1.2) for haplotype phasing of autosomal genotype data, and SHAPEIT2 software (v2.r904) for X-chromosomal genotype data. After phasing, we used Minimac4 software (version 1.0.1) for genome-wide genotype imputation. We used the population-specific imputation reference panel of Japanese individuals (*n* = 1,037) combined with 1000 Genomes Project Phase3v5 samples^22^ (*n* = 2,504). Imputations of the mitochondrial variants were conducted as described elsewhere^40^, using the population-specific reference panel (*n* = 1,037). We applied post-imputation QC filters of MAF ≥ 0.1% and imputation score (Rsq) > 0.5, and obtained 13,116,003, 368,566 and 554 variants for autosomal, X-chromosomal, and mitochondrial variants, respectively. We note that the genotypes of the lead variant in the GWAS (rs60200309) were obtained by imputation (Rsq = 0.88). We assessed accuracy by comparing the imputed dosages with WGS data for the part of the controls (*n* = 236), and confirmed high concordance rate of 97.5%.

### Case–control association test

We conducted GWAS of COVID-19 by using logistic regression of the imputed dosages of each of the variants on case–control status, using PLINK2 software (v2.00a3LM AVX2 Intel (6 July 2020)). We included sex, age, and the top five principal components as covariates in the regression model. We set the genome-wide association significance threshold of *P* < 5.0 × 10^−8^.

### HLA genotype imputation and association test

HLA genotype imputation was performed using DEEP*HLA software (version 1.0), a multitask convolutional deep learning method^14^. We used the population-specific imputation reference panel of Japanese donors (*n* = 1,118), which included both classical and non-classical HLA gene variants for imputation^13^. Before imputation, we removed the overlapping samples between the GWAS controls and the reference panel (*n* = 649), from the GWAS data side. We imputed HLA alleles (two and four digit) and the corresponding HLA amino acid polymorphisms, and applied post-imputation QC filters of MAF ≥ 0.5% and imputation score (*r*^*2*^ in cross-validation) > 0.7.

As for the imputed HLA variants, we conducted (1) association test of binary HLA markers (two- and four-digit HLA alleles) and (2) an omnibus test of each of the HLA amino acid positions, as described elsewhere^13^. Binary maker test was conducted using the same logistic regression model and covariates as in the GWAS. Omnibus test was conducted by a log likelihood ratio test between the null model and the fitted model, followed by a *χ*^2^ distribution with *m* − 1 degrees of freedom, where *m* is the number of residues. *R* statistical software (version 3.6.0) was used for the HLA association test. We set the HLA-wide significance threshold based on Bonferroni’s correction for the number of the HLA tests (*α* = 0.05).

### Estimation of the ABO blood types and analysis

We estimated the ABO blood types of the GWAS subjects based on the five coding variants at the *ABO* gene (rs8176747, rs8176746, rs8176743, rs7853989 and rs8176719)^41^. We phased the haplotypes of these five variants based on the best-guess genotypes obtained by genome-wide imputation, and estimated the ABO blood type as described elsewhere^15^. We were able to unambiguously determine the ABO blood type of 99.1% of the subjects.

Blood-group-specific odds ratios were estimated based on comparisons of A versus AB/B/O, B versus A/AB/O, AB versus A/B/O and O versus A/AB/B. We conducted a logistic regression analysis including age, sex and the top five principal components as covariates. *R* statistical software (version 3.6.3) was used for the ABO blood type analysis.

### Cross-population Mendelian randomization analysis

We conducted two-sample Mendelian randomization analysis as described elsewhere^17,42^. As exposure, we selected a series of clinical states where altered comorbidity with COVID-19 have been discussed. As an outcome phenotype, we used the GWAS summary statistics of Japanese (current study) and European (release 5 from COVID-19 HGI^3^) participants. Lists of the Japanese and European GWAS studies used as the exposure phenotypes are in Supplementary Table 6. We extracted the independent lead variants with genome-wide significance (or the proxy variants in linkage disequilibrium *r*^*2*^ ≥ 0.8 in the EAS or EUR subjects of the 1000 Genomes Project Phase3v5 databases) from the GWAS results of the exposure phenotypes. We applied the inverse variance weighted method using the TwoSampleMR package (version 0.5.5) in *R* statistical software (version 4.0.2).

### Replication analysis

We genotyped additional 1,243 severe COVID-19 cases and 3,769 controls using Infinium Asian Screening Array (Illumina). We applied the QC filters and genotype imputation, and conducted case–control analysis of the variant as in the same manner as the GWAS.

### RNA-seq of peripheral blood of patients with COVID-19

We incorporated 475 patients with COVID-19 recruited at the core medical institutes of JCTF and included them in the GWAS for the bulk RNA-seq analysis (Supplementary Table 2). Isolation of RNA from the peripheral blood of the COVID-19 patients was conducted using RNeasy Mini Kit (Qiagen). Libraries for RNA-seq were prepared using NEBNext Poly(A) mRNA Magnetic Isolation Module and NEBNext Ultra Directional RNA Library Prep Kit for Illumina (New England BioLabs). RNA-seq was performed using the NovaSeq6000 platform (Illumina) with paired-end reads (read length of 100 bp), using S4 Reagent kit (200 cycles). We obtained on average 71,724,142 ± 17,527,007 reads per a sample (mean ± s.d.). Sequencing reads were quality-filtered, and adapter removal was performed using the Trimmomatic (v0.39)^43^. Alignment to the human reference genome GRCh38/hg38 was performed using STAR (v2.7.9a)^44^, based on the GENCODE v30 annotation. Gene level quantification and normalization was using RSEM (v1.3.3)^45^. TPM was used as an index of gene quantification. We excluded the two outlier samples in the principal component analysis plot of the TPM from the analysis (*n* = 473 for the analysis). We quantified 58,825 genes, and adopted the 5,991 genes with median TPM > 10 for the subsequent analysis.

In the eQTL analysis of the *DOCK2* variant, dosage effects of the risk variant (rs60200309-A) on the gene expression levels (TPM) were evaluated using linear regression models with age, sex, severity, the top ten principal components of the TPM matrix, and the top 5 pricipal components of the GWAS data as covariates. The dosage effects of the risk variant on the expression of nearby genes located within a 500-kb window were also evaluated. *R* statistical software (version 3.6.3) was used for the analysis. Colocalization analysis between the GWAS and the *DOCK2* eQTL signals was conducted using eCAVIAR^23^.

### qPCR-based differential expression analysis

Real-time qPCR was conducted for the RNA isolated from the peripheral blood of the COVID-19 patients (*n* = 468). Total RNA was reverse-transcribed using the High-Capacity RNA-to-cDNA cDNA Kit (Life Technologies). Real-time qPCR was performed using TaqMan assays on a 7500 Fast Real-Time PCR system (Applied Biosystems; probe assay ID: Hs00386045_m1 (*DOCK2*) and Hs99999905_m1 (*GAPDH*)). Differential expression analysis was conducted between severe and non-severe COVID-19, and across four COVID-19 disease severity grades, ordered from asymptomatic > mild > severe > most severe. Among the severe COVID-19, patients in intensive care or requiring intubation and ventilation were classed as ‘most severe’ disease, and the rest were classed as ‘severe’ disease. Among the non-severe COVID-19, patients without any symptoms related to COVID-19 were classed as ‘asymptomatic’ disease, and others were classed as ‘mild’ disease. The analysis was performed on relative *DOCK2* mRNA expression relative to *GAPDH* using linear regression models with age and sex as covariates in R statistical software (version 3.6.3).

### Subjects and specimen collection of PBMC for scRNA-seq

Peripheral blood samples were obtained from patients with severe COVID-19 (*n* = 30) and healthy controls (*n* = 31) recruited at Osaka University Graduate School of Medicine. Of the 30 patients with COVID-19, 5 were classed as moderate and 25 were classed as severe according to disease severity based on the highest score on the World Health Organization (WHO) Ordinal Scale for Clinical Improvement. For patients with COVID-19 and healthy controls, blood was collected into heparin tubes and PBMCs were isolated using Leucosep (Greiner Bio-One) density gradient centrifugation according to the manufacturer’s instructions. Blood was processed within 3 h of collection for all samples, and stored at −80 °C until use.

### Droplet-based single-cell sequencing

Single-cell suspensions were processed through the 10x Genomics Chromium Controller (10x Genomics) following the protocol outlined in the Chromium Single Cell V(D)J Reagent Kits (v1.1 Chemistry) User Guide. Chromium Next GEM Single Cell 5′ Library & Gel Bead Kit v1.1 (PN-1000167), Chromium Next GEM Chip G Single Cell Kit (PN-1000127) and Single Index Kit T Set A (PN-1000213) were applied during the process. Approximately 16,500 live cells per sample were separately loaded into each port of the Chromium controller without sample mixing to generate 10,000 single-cell gel-bead emulsions for library preparation and sequencing, according to the manufacturer’s recommendations. Oil droplets of encapsulated single cells and barcoded beads were subsequently reverse-transcribed in a Veriti Thermal Cycler (Thermo Fisher Scientific), resulting in cDNA tagged with a cell barcode and unique molecular index (UMI). Next, cDNA was amplified to generate single-cell libraries according to the manufacturer’s protocol. Quantification was made with an Agilent Bioanalyzer High Sensitivity DNA assay (Agilent, High-Sensitivity DNA Kit, 5067-4626). Subsequently amplified cDNA was enzymatically fragmented, end-repaired, and polyA tagged. Cleanup and size selection was performed on amplified cDNA using SPRIselect magnetic beads (Beckman-Coulter, SPRIselect, B23317). ﻿Next, Illumina sequencing adapters were ligated to the size-selected fragments and cleaned up using SPRIselect magnetic beads. Finally, sample indices were selected and amplified, followed by a double-sided size selection using SPRIselect magnetic beads. Final library quality was assessed using an Agilent Bioanalyzer High Sensitivity DNA assay. Samples were then sequenced on NovaSeq6000 (Illumina) as paired-end mode to achieve a minimum of 20,000 paired-end reads per cell for gene expression.

### Alignment, quantification and QC of scRNA-seq data

Droplet libraries were processed using Cell Ranger 5.0.0 (10x Genomics). Sequencing reads were aligned with STAR (v2.7.2a)^44^ using the GRCh38 human reference genome. Count matrices were built from the resulting BAM files using dropEst^46^. Cells that had fewer than 1,000 UMIs or greater than 20,000 UMIs, as well as cells that contained greater than 10% of reads from mitochondrial or haemoglobin genes, were considered low quality and removed from further analysis. Additionally, putative doublets were removed using Scrublet (v0.2.1) for each sample^47^.

### scRNA-seq computational pipelines and basic analysis

The R package Seurat (v3.2.2) was used for data scaling, transformation, clustering, dimensionality reduction, differential expression analysis and most visualization^48^. Data were scaled and transformed using the SCTransform() function, and linear regression was performed to remove unwanted variation due to cell quality (percentage of mitochondrial reads). ﻿For integration, we identified 3,000 shared highly variable genes (HVGs) using SelectIntegrationFeatures() function. Then, we identified ‘anchors’ between individual datasets based on these genes using the FindIntegrationAnchors() function and inputted these anchors into the IntegrateData() function to create a batch-corrected expression matrix of all cells. Principal component analysis and UMAP dimension reduction with 30 principal components were performed^49^. A nearest-neighbour graph using the 30 dimensions of the principal component analysis reduction was calculated using FindNeighbors() function, followed by clustering using FindClusters() function.

Cellular identity was determined by finding differentially expressed genes for each cluster using FindMarkers() function with parameter ‘test.use=wilcox’, and comparing those markers to known cell-type-specific genes (Extended Data Fig. 7a). We obtained 12 cell clusters, which were further confirmed using Azimuth (Fig. 2d and Extended Data Fig. 7a, c)^50^. Six major cell types were defined from 12 clusters as follows; CD4^+^ T cells and T_reg_ cells were annotated as CD4T; CD8^+^ T cells and proliferative T cells were annotated as CD8T; natural killer cells were annotated as NK; B cells and plasmablasts were annotated as B; CD14^+^monocytes and CD16^+^monocytes were annotated as Mono; conventional dendritic cells and pDCs were annotated as dendritic cells. To clarify immune cell-type-specific expression of *DOCK2*, we produced the density plot using plot_density() function from Nebulosa R package (v1.0.0)^51^, and the dot plot using DotPlot() function.

Droplets labelled as innate immune cell clusters (CD14^+^ monocytes, CD16^+^ monocytes and conventional and pDCs) were extracted and reintegrated for further subclustering using the same procedure as described above except using 2,000 shared HVGs. After integration, clustering and cluster annotation (Extended Data Fig. 7b) were performed as described above.

### Differential expression analysis using scRNA-seq data

Differential gene expression analysis was performed between patients with severe COVID-19 and healthy controls in each cell type. Donor pseudo-bulk samples were first created by aggregating gene counts for each cell type within each sample. Genes which expression rate was more than 10% in either COVID-19 patients or healthy controls in each cell type were included in the analysis. Differential gene expression testing was performed using an NB GLM implemented in the Bioconductor package edgeR (v3.32.0)^52^.

### *DOCK2* co-expression analysis and GO enrichment analysis

We applied the weighted gene co-expression network analysis (WGCNA) algorithm^28^ to evaluate co-expressed genes with *DOCK2* in COVID-19. Pseudo-bulk normalized data of non-classical monocytes in the patients with COVID-19 using scran (v1.18.5)^53^ was used for WGCNA analysis, and genes were selected if they were expressed in more than 1% of cells in non-classical monocytes of the patients with COVID-19. We calculated the adjacency with a ‘unsigned network’ option and soft threshold power with the adjacency matrix set to 5, created Topological Overlap Matrix by TOMsimilarity, calculated the gene tree by hclust against 1 - TOM with method = “average”, and conducted a dynamic tree cut with the following parameters; deepSplit = 4, minClusterSize = 30. We performed GO enrichment analysis of *DOCK2* co-expression gene module using the function enrichGO (pvalueCutoff = 0.01, pAdjustMethod = “BH”, OrgDb = “org.Hs.eg.db”, ont = “BP”) of Clusterprofiler (v3.14.3)^54^.

### Single-cell eQTL analysis of the *DOCK2* risk variant

We applied pseudo-bulk approach for single-cell eQTL analysis. First, we performed single-cell-level normalization using scran (v1.18.5)^53^. Gene expression per cell type per sample was then calculated as the mean of log_2_-transformed counts-per-cell-normalized expression across cells. For principal component analysis, genes were adopted if they were expressed in more than 1% of cells in non-classical monocytes.

In the eQTL analysis of the *DOCK2* variant, dosage effects of the risk variant (rs60200309-A) on the gene expression were evaluated using linear regression models with age, sex, disease severity (included only in COVID-19 analysis) and the top two PCs of the gene expression as covariates. *R* statistical software (version 4.0.2) was used for the analysis.

### IFNα production assay using primary blood cells

PBMC were isolated from the blood of three healthy donors by Lymphoprep density gradient. pDCs were purified by negative selection using the Plasmacytoid Dendritic Cell Isolation Kit II (Miltenyi Biotec). To evaluation interferon-α production ability, sorted pDCs were stimulated with 30 μg ml^−1^ CpG-A ODN (D35; Gene Design, Japan) or control. IFNα was evaluated 12 h after stimulation using VeriKine-HS Human Interferon Alpha All Subtype TCM ELISA Kit (PBL). Differences of IFNα production between the groups were evaluated using paired *t*-test.

### Chemotaxis assay using primary blood cells

PBMC were isolated from the blood of 19 healthy donors by Lymphoprep density gradient. CD3^+^ T cells were sorted by magnetic activated cell sorting (MACS). CD3^+^ T cells (1.0 × 10^5^) in 100 μl RPMI + 0.5% BSA medium ± CPYPP (100 μM; Tocris, UK) were placed in the upper chambers of Transwell (5 µm pore size; Coaster). The lower chambers were filled with 400 µl RPMI medium supplemented with CXCL12 (100 ng ml^−1^; R&D Systems) and incubated at 37 °C for 2 h. The cells that migrated to the lower chambers were collected and analysed using FACS. The following monoclonal antibodies were used for FACS analysis: anti-human CD3 (UCHT1; BD Biosciences) and CD4 (SK3; BD Biosciences) antibodies. Dead cells were excluded using zombie dyes (BioLegend). Events were acquired with a LSR Fortessa (BD Biosciences) and analysed with FlowJo software (BD Biosciences). Differences of chemotaxis between CXCL12 groups and CXCL12 + CPYPP group were evaluated using paired *t*-test.

### *DOCK2* knockdown and IFNα production assay in THP1 Blue ISG cells

THP1-Blue ISG (InvivoGen) cells were cultured in 10% FBS, 2 mM l-glutamine, 25 mM HEPES. To generate lentivirus vectors, LentiCRISPR v2 expressing guide RNA/Cas9 (ref. ^55^), Gag-Pol packaging plasmid psPAX2 (Addgene #12260) and pMD2.G (Addgene #12259) were co-transfected to 293T cells using X-treme GENE 9 DNA Transfection Reagent (Roche). The guide RNA for *DOCK2* knock out and potential off-target effects evaluation^56,57^ were in Supplementary Table 11. Transfected 293T cells were cultured in Dulbecco’s modified Eagle medium with 10% FBS and 50 units per ml penicillin/streptomycin. The cultured medium was replaced 12 h after transfection. The virus-containing supernatants were collected after a further 36 h and filtered through a 0.45-μm pore size cellulose acetate filter (Sigma-Aldrich). Then, 2 × 10^6^ THP1-Blue ISG cells were cultured in 2 ml polybrene (8 µg ml^−1^, Millipore)/virus-containing medium. After a 24 h incubation, infected THP1-Blue ISG cells with virus-containing medium were collected, centrifuged (400*g*, 4 min) and cultured in fresh medium. For selection LentiCRISPR vector expressing cells, infected cells were cultured for 4 days in medium supplemented with 1 μg/ml puromycin 2 days after infection. *DOCK2* knockdown efficiency was evaluated through quantitative real-time PCR analysis and western blotting (Abcam ab124848). THP1 monocytes are differentiated by 72 h incubation with 20 ng ml^−1^ phorbol 12-myristate 13-acetate (PMA, Sigma, P8139). IFNα was evaluated 6 h after stimulation (3 μg ml^−1^ CpG-A ODN (D35, Gene Design) or control ODN (D35, GC)) using VeriKine-HS Human Interferon Alpha All Subtype TCM ELISA Kit (PBL).

### Immunohistochemical analysis of lung samples of patients with COVID-19 pneumonia

Patient samples of lung and hilar lymph node were obtained from autopsies following death from COVID-19 pneumonia (samples 1–3) and non-COVID-19 pneumonia (samples 4 and 5). To stain the control sample, lung and lymph node tissue sections were obtained from the surgically resected lung specimens due to lung cancer. Immunohistochemistry for DOCK2 was performed according to standard procedures. In brief, formalin fixed paraffin embedded tissue sections of 5 μm were deparaffinized. Antigen retrieval was carried out using pressure cooking (in citrate buffer for 3 min). Endogenous peroxidase activity was blocked by incubating sections in 3% hydrogen peroxide for 5 min. After blocking, tissue sections were incubated with the anti-DOCK2 rabbit polyclonal antibody^58^ diluted at 1:1,000. The EnVision kit from Dako (Glostrup) was used to detect the staining.

### In vivo suppression of DOCK2 in Syrian hamster model with SARS-CoV-2 infection

#### Virus

SARS-CoV-2 (JPN/Kanagawa/KUH003)^33^, was used in experimental animal model of COVID-19. An aliquot of virus was stored at −80 °C until use.

#### Materials

CPYPP, an inhibitor of the DOCK2–RAC1 interaction^29^, was obtained from Tocris Bioscience (Bristol, UK). CPYPP was dissolved in DMSO.

#### Animal experiments

All applicable national and institutional guidelines for the care and use of animals were followed. The animal experimentation protocol was approved by the President of Kitasato University through the judgment of the Institutional Animal Care and Use Committee of Kitasato University (approval no. 21-007). Sample sizes were determined based on our experience with SARS-CoV-2 infection models, and the minimum number of animals was used.

### DOCK2 inhibition in a Syrian hamster model of SARS-CoV-2 infection

We planned and executed the experimental schedule shown in Extended Data Fig. 10a. Six-week-old male Syrian hamsters (CLEA Japan) were maintained in the biological safety level 3 experimental animal facility of the Department of Veterinary Medicine, Kitasato University. Sixty-three animals were divided four groups: SARS-CoV-2 + CPYPP (*n* = 29); SARS-CoV-2 + vehicle (*n* = 28); mock + CPYPP (*n* = 3); and mock + vehicle (*n* = 3). Hamsters were intranasally inoculated with 10^5.8^ median tissue culture infectious dose (TCID_50_) of SARS-CoV-2 or medium only (mock infection) in a volume of 100 μl. After 5 min (0 dpi) and 24 h (1 dpi), hamsters were injected intraperitoneally with CPYPP (8.4 mg each; 0.2 ml) or DMSO (vehicle; 0.2 ml). All hamsters were weighed daily. SARS-CoV-2 infected hamsters were euthanized at 3, 6 or 11 dpi (8 animals per group 3 and 6 dpi, and 6 animals per group at 11 dpi), and then nasal swabs and tissues were collected. Lungs were dissected out from thoracic organs after euthanasia, and lung weights were measured at dpi 0, 3, 6 and 11. Differences of body weight and lung weight between SARS-CoV-2+CPYPP group and SARS-CoV-2+vehicle group were evaluated using two-sided Welch’s *t*-test. Hamsters were euthanized when reaching the humane endpoint or 11 days after inoculation with SARS-CoV-2. The humane endpoint (weight loss of > 25%) was based on a previous study^34^.

Syrian hamsters infected with CPYPP or vehicle were euthanized at 3, 6 or 11 dpi for pathological examinations (*n* = 3). Histopathological examination of the lungs of the hamsters inoculated with SARS-CoV-2 with CPYPP or vehicle was conducted by haematoxylin and eosin staining. Pathological severity scores in the infected hamsters were evaluated as described elsewhere^34^. In brief, lung tissue sections were scored based on the percentage of inflammation area of the maximum cut surface collected from each animal in each group by using the following scoring system: 0, no pathological change; 1, affected area (≤10%); 2, affected area (<50%, > 10%); 3, affected area (<90%, ≥50%); 4, (≥90%) an additional point was added when pulmonary oedema and/or alveolar haemorrhage was observed. The total score is shown for individual animals. Immunohistochemistry for alveolar macrophage was performed according to standard procedures. In brief, FFPE lung tissue section of infected Syrian hamster were incubated with the anti-CD68 mouse polyclonal antibody diluted in 1:400 (Abcam ab125212). The EnVision kit (Dako) was used to detect the staining.

Total RNA of nasal swab was extracted using QIAamp Viral RNA Mini kit (Qiagen) according to the manufacturer’s instructions. Each organ was homogenized by adding RLT buffer of QIAamp Viral RNA Mini kit using a multi-bead shocker (Yasui Kikai). After centrifugation of 10% (w/v) tissue homogenate at 10,000 rpm for 10 min, RNA was extracted from the recovered supernatants using the kit described above. The nucleocapsid (*N*) gene of SARS-CoV-2 was detected using THUNDERBIRD Probe One-step qRT-PCR (Toyobo) and Primer/Probe N2 2019-nCoV (TaKaRa). To quantify SARS-CoV-2 N gene copies, a standard curve was generated using Positive Control RNA Mix 2019-nCoV (TaKaRa). Lung cytokine expression profile (IFNs, *Il6* and chemokines) were evaluated with the modifications of Ferren et al.^59^. In brief, 100 ng of RNA was converted to cDNA with the ReverTra Ace qPCR RT Master Mix (Toyobo). qPCR was performed with the THUNDERBIRD Probe qPCR Mix (Toyobo). The primers and probes used are listed in Supplementary Table 12. Reactions for all samples were performed in duplicates using QuantStudio 1 Real-Time PCR System (Thermo Fisher Scientific), and the target mRNA expression levels were normalized with *Gapdh* as a reference gene. Relative expression levels (fold changes) of mRNA from infected hamsters compared with uninfected hamsters were calculated using the 2^−ΔΔ*Ct*^ method with QuantStudio Design and Analysis Software (Thermo Fisher Scientific). Differences of viral load and lung cytokine expression profile between the two groups were evaluated using two-sided Wilcoxon rank sum test.

### Statistics and reproducibility

Figure 2m,n shows representative images of immunohistochemical analysis of DOCK2 in COVID-19 pneumonia and in a control without COVID-19 or pneumonia. Extended Data Fig. 9 shows all of the autopsied cadaver or surgical specimens examined in this study. For immunohistochemical analysis, all experiments were performed on at least three sections of lung and hilar lymph node in each sample, and the similar results were confirmed.

### Reporting summary

Further information on research design is available in the Nature Research Reporting Summary linked to this article.

## Online content

Any methods, additional references, Nature Research reporting summaries, source data, extended data, supplementary information, acknowledgements, peer review information; details of author contributions and competing interests; and statements of data and code availability are available at 10.1038/s41586-022-05163-5.

## Supplementary information

Supplementary Information This file contains Supplementary Figures 1-2 and Supplementary Tables 1–3, 5–9 and 11-12.

Reporting Summary

Supplementary Tables This file contains Supplementary Tables 4 and 10.

## Data Availability

GWAS summary statistics and processed count matrices with differential expression-identified metadata of bulk RNA-seq are deposited at the National Bioscience Database Center (NBDC) Human Database with the accession code hum0343 without restriction. Raw sequencing data of scRNA-seq are available under controlled access at the Japanese Genotype-phenotype Archive (JGA) with accession codes JGAS000543 and JGAD000662 for general research use, which can be accessed through application at the NBDC with the accession code hum0197. GWAS genotype data of the COVID-19 cases are available under controlled access at European Genome-Phenome Archive (EGA) with the accession code EGAS00001006284 for general research use. GWAS genotype data of the controls collected at Osaka University and the affiliated medical institutes are available under controlled access at EGA with the accession code EGAS00001006423 for use as controls. GWAS genotype data of the controls collected at University of Tsukuba cannot be deposited, since no consent was obtained for deposition in a public repository, but these data are available upon request (nhizawa@md.tsukuba.ac.jp) for use as controls in research of inflammatory lung disease. The GWAS summary statistics of COVID-19 HGI (release 5) were obtained from https://www.covid19hg.org/results/r5/. The reference for cell-type annotation of PBMC in scRNA-seq (pbmc_multimodal.h5seurat) was obtained from https://satijalab.org/seurat/articles/multimodal_reference_mapping.html.
