# Supplementary material for: DOCK2 is involved in the host genetics and biology of severe COVID-19
Source: Nature. 2022 Aug 8;609(7928):754–60. doi: 10.1038/s41586-022-05163-5 (PMC9492544; doi:10.1038/s41586-022-05163-5)
Supplement: Supplementary file 1 — This file contains Supplementary Figures 1-2 and Supplementary Tables 1–3, 5–9 and 11-12. [file 41586_2022_5163_MOESM1_ESM.pdf]

nature portfolio

https://doi.org/10.1038/s41586-022-05163-5

Supplementary information

DOCK2 is involved in the host genetics and biology of severe COVID-19

In the format provided by the authors and unedited

Supplementary Information

DOCK2 is involved in the host genetics and biology of severe COVID-19

The Japan COVID-19 Task Force.

Corresponding to:
Koichi Fukunaga (kfukunaga@keio.jp), Division of Pulmonary Medicine, Department of Medicine, Keio University School of Medicine, Tokyo, Japan.
Yukinori Okada (yokada@sg.med.osaka-u.ac.jp), Department of Statistical Genetics, Osaka University Graduate School of Medicine, Suita, Japan.

Table of Contents

Supplementary Figure …………………………………………………………………. 2

1. Biological impact of the *DOCK2* risk variant in primary cells …………………………... 2
2. Knockdown of *DOCK2* by CRISPR system ……………………………………………… 3

Supplementary Table ………………………………………………………………….. 4

1. A list of the medical institutes participating to Japan COVID-19 Task Force……………………... 4
2. Characteristics of the study participants ………………………………………….……. 7
3. Associations of the previously reported COVID-19 risk variants ………...……………..... 9
4. HLA variant associations with COVID-19 risk ……………………………………….... 10
5. Associations of the ABO blood type with COVID-19 risk ……………………………… 11
6. Details of the GWAS studies of the exposure phenotypes in the Mendelian randomization analysis ...……………………………………………………………………………... 13
7. Results of the cross-population Mendelian randomization analysis on COVID-19 …… 14
8. Detailed associations of the *DOCK2* rs60200309-A allele with COVID-19 susceptibility ………………………………………………………………………………………. 15
9. A replication study of the *DOCK2* variant in the pan-ancestry meta-analysis ………… 16
10. eQTL analysis result of the variants around the *DOCK2* region ………..……………… 17
11. The guide RNA for *DOCK2* knockdown used in LentiCRISPR system and sgRNA sequence annotation ……………………………………………………………………… 18
12. The primers and probes used for evaluation of lung cytokine expression profile in SARS-CoV-2 infected Syrian hamster ……………………………………………………….... 19

Supplementary Figure 1. Biological impact of the *DOCK2* risk variant in primary cells

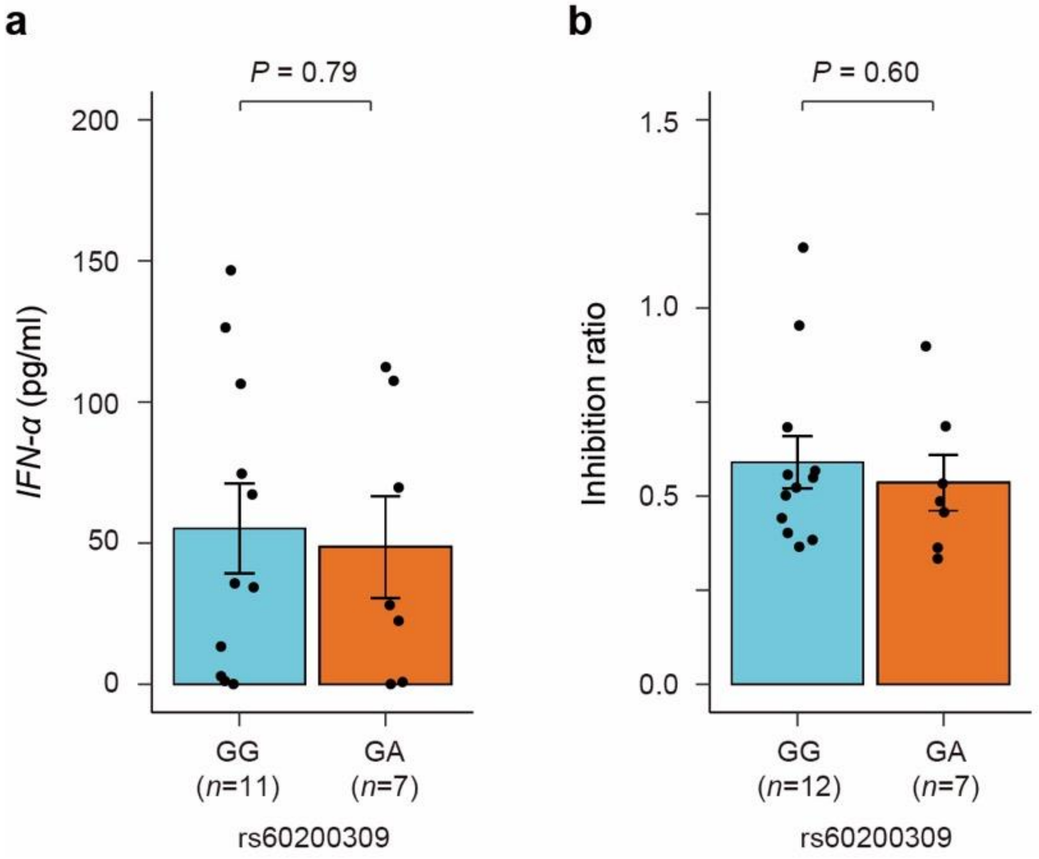

(**a**) The impact of the *DOCK2* risk variant on interferon-α (*IFN-α*) production ability in pDC. Sorted pDC were stimulated with CpG. (**b**) The impact of the *DOCK2* risk variant on chemotaxis in $CD3^+$ T cells. $CD3^+$ T cells were stimulated with CXCL12 or CXCL12 + CPYPP, and inhibition ratio by CPYPP was estimated. In (**a**) and (**b**), data shows mean + s.e.m., and differences between the groups were evaluated using two-sided Welch's *t*-test.

Supplementary Figure 2. Knockdown of *DOCK2* by CRISPR system

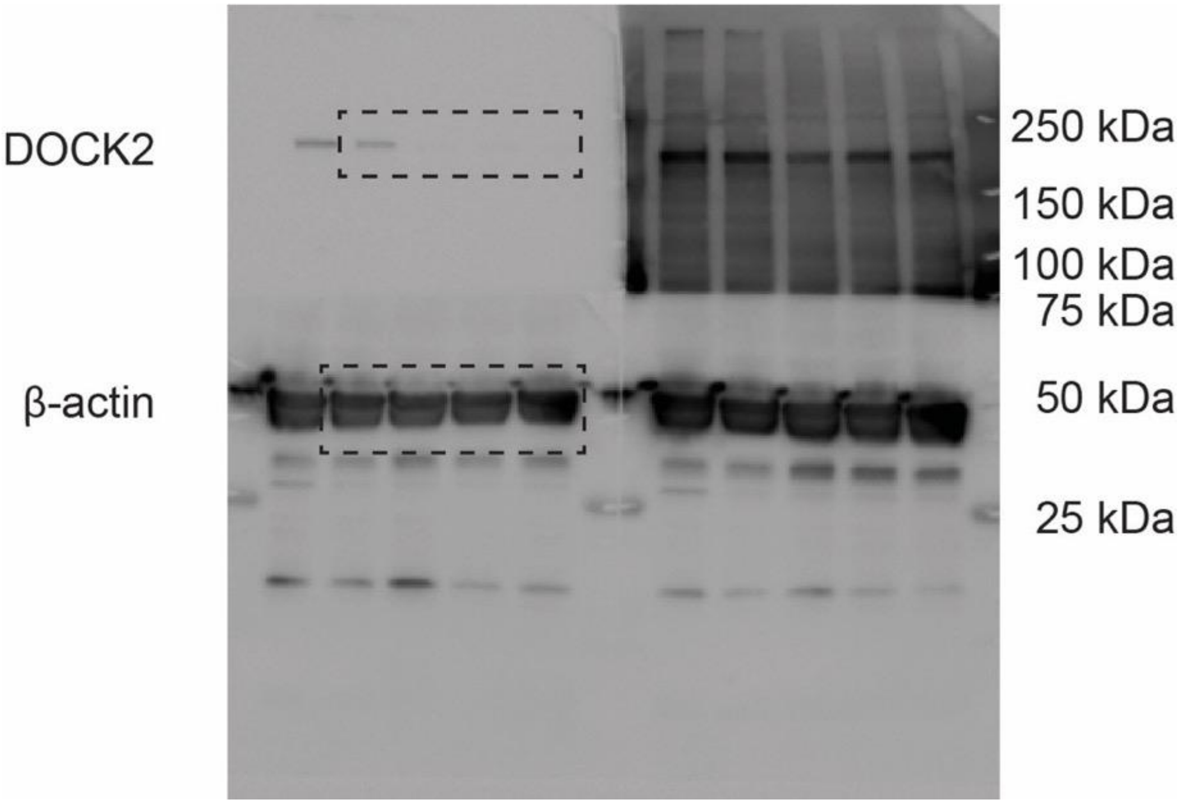

Full size image from **Extended Data Fig. 8c**.

Supplementary Table 1. A list of the medical institutes participating to Japan COVID-19 Task Force

Aichi Cancer Center Hospital
Chiba University Graduate School of Medicine
Daini Osaka Police Hospital
Eiju General Hospital
Faculty of Medicine
Fujioka General Hospital
Fujisawa City Hospital
Fukujuji hospital
Fukuoka Tokushukai Hospital
Fukuoka University
Fukuoka University Hospital
Fukushima Medical University
Gifu University School of Medicine Graduate School of Medicine
Graduate School of Tokyo Institute of Technology
Gunma University Graduate School of Medicine
Himeji St. Mary's Hospital
International University of Health and Welfare Shioya Hospital
Ishikawa Prefectural Central Hospital
JA Toride medical hospital
Japan Community Health care Organization Kanazawa Hospital
Japanese Red Cross Medical Center
JCHO (Japan Community Health care Organization) Saitama Medical Center
Juntendo University Graduate School of Medicine
Kanagawa Cardiovascular and Respiratory Center
Kansai Electric Power Hospital
Kansai Medical University General Medical Center
Kansai Rosai Hospital
Kanto Rosai Hospital
Kawasaki Municipal Ida Hospital
Keio University Hospital
Keio University School of Medicine
Keiyu Hospital
KINSHUKAI Hanwa The Second Hospital
Kiryu Kosei General Hospital
Kitasato University
Kitasato University Kitasato Institute Hospital
KKR Sapporo Medical Center
Kobe University Graduate School of Medicine
Kumamoto City Hospital
Kurume University School of Medicine
Kyoto Prefectural University of Medicine

Kyoto University Graduate School of Medicine
Kyushu University Graduate School of Medical Sciences
Matsumoto City Hospital
Musashino Red Cross Hospital
Nagoya University Graduate School of Medicine
National Center for Global Health and Medicine
National Defense Medical College
National Hospital Organization Hokkaido Medical Center
National Hospital Organization Kumamoto Medical Center
National Hospital Organization Kyoto Medical Center
National Hospital Organization Kyushu Medical Center
National hospital organization Saitama Hospital
National Hospital Organization Tokyo Hospital
National Hospital Organization Tokyo Medical Center
NHO Kanazawa Medical Center
Nihon University School of Medicine
Niigata University
Okayama Rosai Hospital
Ome Municipal General Hospital
Osaka Saiseikai Nakatsu Hospital
Osaka University Graduate School of Medicine
Osaka University Hospital
Saiseikai Kumamoto Hospital
Saiseikai Utsunomiya Hospital
Saiseikai Yokohamashi Nanbu Hospital
Saitama Cardiovascular and Respiratory Center
Saitama City Hospital
Sano Kosei General Hospital
Sapporo City General Hospital
Showa University
Showa University Koto Toyosu Hospital
St Marianna University School of Medicine, Yokohama-City Seibu Hospital
St. Marianna University School of Medicine
Tachikawa Hospital
The Institute of Medical Science, the University of Tokyo
Toho University Ohashi Medical Center
Tohoku University Graduate School of Medicine
Tokai University School of Medicine
Tokyo Medical and Dental University
Tokyo Medical and Dental University Hospital of Medicine
Tokyo Medical University Hospital
Tokyo Medical University Ibaraki Medical Center
Tokyo Metropolitan Police Hospital
Tokyo Saiseikai Central Hospital

Tokyo Women's Medical University Medical Center East
Tokyo Medical and Dental University Hospital of Medicine
Tosei General Hospital
Toyohashi Municipal Hospital
Tsukuba Kinen General Hospital
Uji-Tokushukai Medical Center
University of Tsukuba
Yamagata University Faculty of Medicine

Medical institutes and hospitals contributing to the current GWAS study are listed.

Supplementary Table 2. Characteristics of the study participants

GWAS participants

Subjects	Age	Severity	No. subjects	Age (mean±SD)	Male proportion (%)
COVID-19 cases*	All age	All	2,393	56.0 ± 18.9	64.2
		Severe	990	65.3 ± 13.9	73.9
		Non-severe	1,391	49.3 ± 19.2	57.2
	Age < 65	All	1,484	44.1 ± 13.3	65.7
		Severe	440	52.6 ± 9.2	81.8
		Non-severe	1,041	40.5 ± 13.1	58.9
	Age ≥ 65	All	909	75.4 ± 7.1	61.7
		Severe	550	75.5 ± 6.9	67.6
		Non-severe	350	75.2 ± 7.6	52.3
Controls	All Age	-	3,289	53.1 ± 16.8	47.5
	Age < 65		2,377	45.1 ± 12.1	47.8
	Age ≥ 65		912	74.0 ± 6.2	46.5

* COVID-19 cases were collected from April 2020 to January 2021.

Replication participants

Subjects	Age	Severity	No. subjects	Age (mean±SD)	Male proportion (%)
COVID-19 cases*	All Age	Severe	1,243	58.4 ± 14.4	71.8
	Age < 65		833	50.3 ± 9.6	76.1
	Age ≥ 65		410	74.8 ± 6.5	62.9
Controls	All Age	-	3,769	67.1 ± 13.4	59.0
	Age < 65		1,242	52.0 ± 11.0	50.1
	Age ≥ 65		2,527	74.6 ± 6.2	63.4

* COVID-19 cases were collected from February 2021 to September 2021.

Baseline characteristics of GWAS and replication participants

Cohort	GWAS participants				Replication participants	
Characteristics	All age (*n* = 2,393)	All age and Severe (*n* = 990)	Age < 65 (*n* = 1,484)	Age < 65 and Severe (*n* = 440)	All age and Severe (*n* = 1,243)	Age < 65 and Severe (*n* = 833)
Body-mass index	23.7 (21.4-26.7)	24.7 (22.2-27.5)	24.2 (21.8-27.4)	26.6 (24.2-30.0)	25.7 (22.9-29.0)	26.6 (23.8-29.9)
Smoking status (Never)	860 (52.7)	313 (48.2)	527 (53.0)	128 (44.0)	376 (48.5)	234 (48.0)
Comorbidities						
Hypertension	592 (33.6)	344 (47.9)	208 (19.5)	109 (34.6)	347 (41.8)	169 (32.7)
Diabetes mellitus	391 (22.2)	232 (32.4)	172 (16.0)	93 (29.2)	212 (25.5)	123 (23.8)
Hyperuricemia	186 (10.6)	98 (13.9)	106 (9.9)	53 (17.1)	96 (11.5)	51 (9.8)
Cardiovascular disease	180 (10.2)	109 (15.2)	46 (4.3)	25 (7.9)	94 (11.3)	29 (5.6)
Malignancy	122 (7.0)	63 (8.9)	41 (3.8)	18 (5.8)	52 (6.3)	14 (2.7)
Autoimmune disease	67 (3.8)	35 (4.9)	38 (3.5)	18 (5.7)	29 (3.5)	15 (2.9)
COPD	77 (4.4)	59 (8.3)	28 (2.6)	21 (6.6)	42 (5.1)	11 (2.1)
Asthma	121 (7.0)	42 (6.0)	79 (7.5)	26 (8.4)	56 (6.8)	38 (7.5)
Chronic liver disease	67 (3.9)	32 (4.6)	46 (4.4)	19 (6.3)	40 (4.9)	25 (4.9)
Chronic kidney disease	113 (6.7)	77 (11.1)	39 (3.8)	18 (6.0)	76 (9.4)	28 (5.5)

Data are median (interquartile range) or number (%). COPD=chronic obstructive pulmonary disease.

Bulk RNA-seq participants

Age	Severity	No. subjects	Age (mean±SD)	Male proportion (%)
All age	All	473	59.6 ± 17.7	67.9
	Severe	368	64.3 ± 14.8	70.9
	Non-severe	105	42.9 ± 17.2	57.1
Age < 65	All	270	47.4 ± 12.8	74.1
	Severe	179	52.0 ± 9.8	81.0
	Non-severe	91	38.4 ± 13.2	60.4
Age ≥ 65	All	203	75.8 ± 7.4	59.6
	Severe	189	76.0 ± 7.4	61.4
	Non-severe	14	72.8 ± 6.4	35.7

Supplementary Table 3. Associations of the previously reported COVID-19 risk variants

rsID	Chr:position (b37)	Allele Risk/non-risk	Gene	Phenotype	Risk allele frequency Case	Control	OR (95%CI)	*P*
rs2271616	3:45838013	T/G	*SLC6A20*	COVID-19 vs control	0.128	0.129	0.98 (0.87-1.10)	0.75
				Severe COVID-19 vs control	0.129	0.129	0.97 (0.81-1.14)	0.68
				COVID-19 vs control (age<65)	0.129	0.129	1.01 (0.87-1.16)	0.92
				Severe COVID-19 vs control (age<65)	0.134	0.129	0.97 (0.77-1.23)	0.79
rs35081325	3:45889921	T/A	*LZTFL1*	COVID-19 vs control	0.0021	0.0013	2.49 (0.77-8.08)	0.13
				Severe COVID-19 vs control	0.0032	0.0013	**7.06 (1.63-30.6)**	**0.0090**
				COVID-19 vs control (age<65)	0.0021	0.0013	2.22 (0.53-9.39)	0.28
				Severe COVID-19 vs control (age<65)	0.0030	0.0013	**11.8 (1.64-85.5)**	**0.014**
rs11919389	3:101424458	T/C	*RPL24*	COVID-19 vs control	0.626	0.625	1.02 (0.94-1.10)	0.66
				Severe COVID-19 vs control	0.619	0.625	1.02 (0.91-1.14)	0.79
				COVID-19 vs control (age<65)	0.623	0.632	0.97 (0.88-1.07)	0.56
				Severe COVID-19 vs control (age<65)	0.615	0.632	0.95 (0.81-1.12)	0.57
rs1886814	6:41502683	C/A	*FOXP4*	COVID-19 vs control	0.307	0.285	**1.14 (1.04-1.24)**	**0.0035**
				Severe COVID-19 vs control	0.317	0.285	**1.29 (1.13-1.46)**	**9.1×10^{-5}**
				COVID-19 vs control (age<65)	0.314	0.285	**1.17 (1.05-1.30)**	**0.0045**
				Severe COVID-19 vs control (age<65)	0.340	0.285	**1.42 (1.19-1.69)**	**1.2×10^{-4}**
rs72711165	8:125336564	C/T	*TMEM65*	COVID-19 vs control	0.035	0.031	1.15 (0.93-1.42)	0.21
				Severe COVID-19 vs control	0.034	0.031	1.15 (0.85-1.57)	0.37
				COVID-19 vs control (age<65)	0.040	0.030	**1.31 (1.01-1.70)**	**0.040**
				Severe COVID-19 vs control (age<65)	0.044	0.030	1.42 (0.94-2.16)	0.094
rs529565	9:136149500	C/T	*ABO*	COVID-19 vs control	0.475	0.456	1.08 (1.00-1.16)	0.057
				Severe COVID-19 vs control	0.494	0.456	**1.18 (1.06-1.32)**	**0.0028**
				COVID-19 vs control (age<65)	0.476	0.458	1.08 (0.99-1.19)	0.089
				Severe COVID-19 vs control (age<65)	0.510	0.458	**1.22 (1.04-1.42)**	**0.012**
rs10774671	12:113357193	A/G	*OAS1*	COVID-19 vs control	0.800	0.791	1.06 (0.96-1.17)	0.24
				Severe COVID-19 vs control	0.802	0.791	1.08 (0.94-1.24)	0.30
				COVID-19 vs control (age<65)	0.805	0.794	1.07 (0.95-1.20)	0.28
				Severe COVID-19 vs control (age<65)	0.814	0.794	1.09 (0.89-1.34)	0.38
rs1819040	17:44219831	T/A	*KANSL1*	Not imputable				
rs77534576	17:47940666	T/C	*TAC4*	COVID-19 vs control	0.048	0.045	1.07 (0.89-1.29)	0.46
				Severe COVID-19 vs control	0.057	0.045	**1.29 (1.01-1.65)**	**0.043**
				COVID-19 vs control (age<65)	0.051	0.044	1.16 (0.93-1.45)	0.20
				Severe COVID-19 vs control (age<65)	0.060	0.044	1.33 (0.95-1.88)	0.098
rs2109069	19:4719443	A/G	*DPP9*	COVID-19 vs control	0.123	0.111	1.10 (0.98-1.24)	0.12
				Severe COVID-19 vs control	0.133	0.111	**1.20 (1.02-1.42)**	**0.031**
				COVID-19 vs control (age<65)	0.120	0.113	1.06 (0.91-1.22)	0.46
				Severe COVID-19 vs control (age<65)	0.131	0.113	1.11 (0.88-1.41)	0.39
rs74956615	19:10427721	A/T	*RAVER1*	Not imputable				
rs4801778	19:49370609	G/T	*PLEKHA4*	COVID-19 vs control	0.978	0.975	1.15 (0.88-1.49)	0.30
				Severe COVID-19 vs control	0.977	0.975	1.06 (0.73-1.54)	0.77
				COVID-19 vs control (age<65)	0.978	0.973	1.23 (0.89-1.69)	0.20
				Severe COVID-19 vs control (age<65)	0.980	0.973	1.29 (0.74-2.24)	0.37
rs13050728	21:34615210	T/C	*IFNAR2*	COVID-19 vs control	0.573	0.549	**1.10 (1.02-1.19)**	**0.015**
				Severe COVID-19 vs control	0.572	0.549	1.08 (0.97-1.22)	0.17
				COVID-19 vs control (age<65)	0.580	0.543	**1.16 (1.06-1.28)**	**0.0024**
				Severe COVID-19 vs control (age<65)	0.605	0.543	**1.28 (1.08-1.50)**	**0.0039**

Uncorrected *P* values are shown. Associations with $P < 0.05$ are highlighted in bold.

Supplementary Table 4. HLA variant associations with COVID-19 risk

(Results are indicated in a separate Microsoft Excel file)

Supplementary Table 5. Associations of the ABO blood type with COVID-19 risk

Associations of the target blood type compared with other blood types

Age	Phenotype	Target vs other blood types	Target blood type No. and freq.				OR (95%CI)	*P*
			N_{cases}	$N_{controls}$	$freq_{Cases}$	$freq_{controls}$		
All age	COVID-19 vs control	A vs AB/B/O	974	1,274	0.411	0.390	1.10 (0.98-1.23)	0.093
		B vs A/AB/O	505	712	0.213	0.218	0.95 (0.83-1.09)	0.46
		AB vs A/B/O	251	310	0.106	0.095	1.13 (0.95-1.35)	0.18
		O vs A/AB/B	639	968	0.270	0.297	**0.88 (0.78-1.00)**	**0.041**
	Severe COVID-19 vs control	A vs AB/B/O	398	1,274	0.407	0.390	1.04 (0.89-1.22)	0.61
		B vs A/AB/O	216	712	0.221	0.218	1.00 (0.83-1.21)	0.96
		AB vs A/B/O	117	310	0.120	0.095	**1.41 (1.10-1.81)**	**0.0065**
		O vs A/AB/B	248	968	0.253	0.297	**0.81 (0.68-0.96)**	**0.016**
Age < 65	COVID-19 vs control	A vs AB/B/O	607	900	0.412	0.381	**1.18 (1.03-1.35)**	**0.017**
		B vs A/AB/O	315	524	0.214	0.222	0.92 (0.78-1.08)	0.33
		AB vs A/B/O	161	235	0.109	0.099	1.09 (0.88-1.36)	0.41
		O vs A/AB/B	390	703	0.265	0.298	**0.85 (0.73-0.98)**	**0.027**
	Severe COVID-19 vs control	A vs AB/B/O	173	900	0.394	0.381	1.08 (0.86-1.35)	0.52
		B vs A/AB/O	103	524	0.235	0.222	1.06 (0.81-1.37)	0.67
		AB vs A/B/O	60	235	0.137	0.099	**1.40 (1.00-1.94)**	**0.048**
		O vs A/AB/B	103	703	0.235	0.298	**0.73 (0.56-0.93)**	**0.014**

Associations of the target blood type compared with the O blood type

Age	Phenotype	Target blood types vs O	Target blood type freq.		OR (95%CI)	*P*
			Cases	control		
All age	COVID-19 vs control	A vs O	0.411	0.390	**1.16 (1.02-1.32)**	**0.029**
		B vs O	0.213	0.218	1.05 (0.90-1.23)	0.52
		AB vs O	0.106	0.095	**1.22 (1.00-1.48)**	**0.050**
	Severe COVID-19 vs control	A vs O	0.407	0.390	1.20 (0.99-1.46)	0.068
		B vs O	0.221	0.218	1.17 (0.93-1.46)	0.18
		AB vs O	0.120	0.095	**1.57 (1.19-2.08)**	**0.0015**
Age < 65	COVID-19 vs control	A vs O	0.412	0.381	**1.25 (1.06-1.48)**	**0.0074**
		B vs O	0.214	0.222	1.06 (0.88-1.28)	0.55
		AB vs O	0.109	0.099	1.23 (0.96-1.56)	0.097
	Severe COVID-19 vs control	A vs O	0.394	0.381	1.32 (0.99-1.76)	0.057
		B vs O	0.235	0.222	1.32 (0.96-1.82)	0.084
		AB vs O	0.137	0.099	**1.67 (1.14-2.44)**	**0.0081**

Associations of the target blood type compared with the A blood type

Age	Phenotype	Target blood types vs A	Target blood type freq.		OR (95%CI)	*P*
			Cases	control		
All age	COVID-19 vs control	B vs A	0.213	0.218	0.91 (0.79-1.05)	0.21
		AB vs A	0.106	0.095	1.06 (0.87-1.28)	0.57
		O vs A	0.270	0.297	**0.86 (0.76-0.98)**	**0.029**
	Severe COVID-19 vs control	B vs A	0.221	0.218	0.98 (0.80-1.20)	0.85
		AB vs A	0.120	0.095	**1.34 (1.03-1.75)**	**0.029**
		O vs A	0.253	0.297	0.83 (0.69-1.01)	0.068
Age < 65	COVID-19 vs control	B vs A	0.214	0.222	0.85 (0.71-1.02)	0.079
		AB vs A	0.109	0.099	0.98 (0.78-1.23)	0.87
		O vs A	0.265	0.298	**0.80 (0.68-0.94)**	**0.0074**
	Severe COVID-19 vs control	B vs A	0.235	0.222	1.01 (0.75-1.35)	0.96
		AB vs A	0.137	0.099	1.34 (0.93-1.91)	0.12
		O vs A	0.235	0.298	0.76 (0.57-1.01)	0.057

Associations of the target blood type compared with the B blood type

Age	Phenotype	Target blood types vs B	Target blood type freq.		OR (95%CI)	*P*
			Cases	control		
All age	COVID-19 vs control	A vs B	0.411	0.390	1.10 (0.95-1.27)	0.21
		AB vs B	0.106	0.095	1.16 (0.94-1.43)	0.16
		O vs B	0.270	0.297	0.95 (0.81-1.11)	0.52
	Severe COVID-19 vs control	A vs B	0.407	0.390	1.02 (0.83-1.26)	0.85
		AB vs B	0.120	0.095	**1.35 (1.01-1.80)**	**0.041**
		O vs B	0.253	0.297	0.86 (0.69-1.07)	0.18
Age < 65	COVID-19 vs control	A vs B	0.412	0.381	1.17 (0.98-1.40)	0.079
		AB vs B	0.109	0.099	1.15 (0.89-1.47)	0.29
		O vs B	0.265	0.298	0.94 (0.78-1.14)	0.55
	Severe COVID-19 vs control	A vs B	0.394	0.381	0.99 (0.74-1.33)	0.96
		AB vs B	0.137	0.099	1.25 (0.85-1.84)	0.26
		O vs B	0.235	0.298	0.76 (0.55-1.04)	0.084

Associations of the target blood type compared with the A or B blood types

Age	Phenotype	Target blood types vs A or B	Target blood type freq.		OR (95%CI)	*P*
			Cases	control		
All age	COVID-19 vs control	AB vs A or B	0.106	0.095	1.09 (0.91-1.31)	0.34
		O vs A or B	0.270	0.297	0.89 (0.79-1.01)	0.072
	Severe COVID-19 vs control	AB vs A or B	0.120	0.095	**1.34 (1.04-1.73)**	**0.023**
		O vs A or B	0.253	0.297	0.84 (0.70-1.01)	0.058
Age < 65	COVID-19 vs control	AB vs A or B	0.109	0.099	1.04 (0.83-1.30)	0.73
		O vs A or B	0.265	0.298	**0.85 (0.73-0.99)**	**0.035**
	Severe COVID-19 vs control	AB vs A or B	0.137	0.099	1.29 (0.91-1.81)	0.14
		O vs A or B	0.235	0.298	**0.75 (0.58-0.98)**	**0.034**

Uncorrected *P* values are shown. Associations with *P* < 0.05 are highlighted in bold.

Supplementary Table 6. Details of the GWAS studies of the exposure phenotypes in the Mendelian randomization analysis

Population	Phenotype	No. subjects			Reference	PMID
		Cases	Controls	Total		
Japanese	Body mass index (BMI)	-	-	173,430	Akiyama et al. *Nat Genet* (2017)	28892062
	Type 2 diabetes (T2D)	77,418	356,122	433,540	Spracklen et al. *Nature* (2020)	32499647
	Cigarettes per day (CPD)	-	-	72,655	Matoba et al. Nat Hum Behav(2019)	31089300
	Asthma	8,216	201,592	209,808	Ishigaki et al. *Nat Genet* (2020)	32514122
	Systolic blood pressure (sBP)	-	-	183,785	Takeuchi et al. *Nat Commun* (2018)	30487518
	Diastolic blood pressure (dBP)	-	-	183,785	Takeuchi et al. *Nat Commun* (2018)	30487518
	Estimated glomerular filtration rate (eGFR)	-	-	143,658	Kanai et al. *Nat Genet* (2018)	29403010
	Serum uric acids (UA)	-	-	121,745	Nakatochi et al. *Commun Biol* (2019)	30993211
	Gout	3,053	4,554	7,607	Nakayama et al. *Ann Rheum Dis* (2020)	32238385
	Rheumatoid arthritis (RA)	4,873	17,641	22,514	Okada et al. *Nature* (2014)	24390342
	Systemic lupus erythematosus (SLE)	13,377	194,993	208,370	Yin et al. *Ann Rheum Dis* (2020)	33272962
European	Body mass index (BMI)	-	-	681,275	Yengo et al. *Hum Mol Genet* (2018)	30124842
	Type 2 diabetes (T2D)	148,726	965,732	1,114,458	Vujkovic et al. *Nat Gene*t (2020)	32541925
	Cigarettes per day (CPD)	-	-	337,334	Liu et al. Nat Genet (2019)	30643251
	Asthma	64,538	329,321	393,859	Han et al. *Nat Commun* (2020)	32296059
	Systolic blood pressure (sBP)	-	-	757,601	Evangelou et al. *Nat Genet* (2018)	30224653
	Diastolic blood pressure (dBP)	-	-	757,601	Evangelou et al. *Nat Genet* (2018)	30224653
	Estimated glomerular filtration rate (eGFR)	-	-	567,460	Wuttke et al. *Nat Genet* (2019)	31152163
	Serum uric acids (UA)	-	-	288,649	Tin et al. *Nat Genet* (2019)	31578528
	Gout	13,179	750,634	763,813	Tin et al. *Nat Genet* (2019)	31578528
	Rheumatoid arthritis (RA)	14,361	43,923	58,284	Okada et al. *Nature* (2014)	24390342
	Systemic lupus erythematosus (SLE)	5,201	9,066	14,267	Bentham et al. *Nat Genet* (2015)	26502338

Supplementary Table 7. Results of the cross-population Mendelian randomization analysis on COVID-19

Exposure phenotypes (Japanese)	COVID-19 vs control				Severe COVID-19 vs control				COVID-19 vs control (age<65)				Severe COVID-19 vs control (age<65)			
	N_{SNP}	Beta	SE	*P*	N_{SNP}	Beta	SE	*P*	N_{SNP}	Beta	SE	*P*	N_{SNP}	Beta	SE	*P*
Body mass index (BMI)	76	0.296	0.173	0.087	76	**0.676**	**0.249**	**0.0067**	76	0.380	0.213	0.075	76	**0.946**	**0.353**	**0.0074**
Type 2 diabetes (T2D)	164	0.050	0.048	0.30	164	0.075	0.069	0.27	164	0.032	0.059	0.59	164	0.069	0.099	0.48
Cigarettes per day (CPD)	5	0.167	0.310	0.59	5	-0.236	0.449	0.60	5	0.015	0.381	0.97	5	-0.887	0.637	0.16
Asthma	7	**0.376**	**0.137**	**0.0061**	7	0.165	0.197	0.40	7	**0.401**	**0.169**	**0.018**	7	0.195	0.279	0.49
Systolic blood pressure (sBP)	54	0.011	0.011	0.35	54	-0.008	0.016	0.62	54	0.014	0.014	0.31	54	-0.022	0.023	0.33
Diastolic blood pressure (dBP)	41	0.017	0.021	0.42	41	-0.022	0.030	0.46	41	0.014	0.025	0.57	41	-0.051	0.042	0.23
Estimated glomerular filtration rate (eGFR)	67	-0.070	0.170	0.68	67	0.082	0.245	0.74	67	0.027	0.209	0.90	67	0.147	0.347	0.67
Serum uric acids (UA)	36	0.163	0.095	0.088	36	0.024	0.137	0.86	36	**0.279**	**0.119**	**0.019**	36	0.216	0.199	0.28
Gout	10	**0.078**	**0.028**	**0.0048**	10	0.018	0.039	0.65	10	**0.104**	**0.035**	**0.0027**	10	0.059	0.057	0.30
Rheumatoid arthritis (RA)	15	-0.065	0.048	0.18	15	-0.018	0.070	0.80	15	-0.102	0.059	0.083	15	-0.067	0.097	0.49
Systemic lupus erythematosus (SLE)	100	**-0.087**	**0.027**	**0.0014**	100	-0.060	0.039	0.12	100	-0.058	0.033	0.084	100	0.022	0.056	0.70

Exposure phenotypes (European)	Self-reported COVID-19 (C2)				Hospitalized COVID-19 (B2)				Severe COVID-19 (A2)			
	N_{SNP}	Beta	SE	*P*	N_{SNP}	Beta	SE	*P*	N_{SNP}	Beta	SE	*P*
Body mass index (BMI)	486	**0.175**	**0.030**	**8.5×10^{-9}**	486	**0.398**	**0.060**	**3.2×10^{-11}**	486	**0.405**	**0.090**	**6.2×10^{-6}**
Type 2 diabetes (T2D)	414	**0.027**	**0.012**	**0.019**	413	**0.061**	**0.023**	**0.0078**	417	0.068	0.035	0.050
Cigarettes per day (CPD)	39	0.032	0.067	0.63	39	0.255	0.132	0.054	39	0.096	0.199	0.63
Asthma	124	-0.026	0.017	0.14	119	-0.025	0.035	0.48	124	-0.059	0.052	0.26
Systolic blood pressure (sBP)	396	-0.0015	0.0018	0.41	394	0.0019	0.0036	0.59	396	-0.0036	0.0055	0.51
Diastolic blood pressure (dBP)	408	-0.0038	0.0030	0.20	405	-0.0021	0.0061	0.72	407	-0.011	0.009	0.25
Estimated glomerular filtration rate (eGFR)	256	0.014	0.211	0.95	255	-0.459	0.423	0.28	256	**-1.315**	**0.649**	**0.043**
Serum uric acids (UA)	121	-0.013	0.019	0.51	121	0.028	0.038	0.46	122	0.048	0.056	0.39
Gout	27	-0.007	0.012	0.58	27	-0.020	0.024	0.42	28	-0.009	0.036	0.81
Rheumatoid arthritis (RA)	63	-0.010	0.011	0.37	63	-0.028	0.022	0.20	63	0.028	0.033	0.40
Systemic lupus erythematosus (SLE)	41	-0.011	0.007	0.11	41	-0.016	0.014	0.25	41	-0.024	0.020	0.24

Uncorrected *P* values are shown. Associations with $P < 0.05$ are highlighted in bold.

Supplementary Table 8. Detailed associations of the *DOCK2* rs60200309-A allele with COVID-19 susceptibility

Recruitment period	Age	Phenotype	No. subjects		Risk allele frequency (A)		OR (95%CI)	*P*
			Cases	Controls	Cases	Controls		
April 2020 - Jan 2021	All age	COVID-19 vs control	2,393	3,289	0.119	0.101	1.24 (1.09-1.41)	0.0011
		Severe COVID-19 vs control	990	3,289	0.129	0.101	1.39 (1.16-1.66)	3.1×10^{-4}
		Non-Severe COVID-19 vs control	1,391	3,289	0.113	0.101	1.16 (1.00-1.35)	0.054
		Severe COVID-19 vs Non-Severe COVID-19	990	1,391	0.129	0.113	1.27 (1.03-1.57)	0.028
	Age < 65	COVID-19 vs control	1,484	2,377	0.123	0.099	1.32 (1.13-1.55)	5.1×10^{-4}
		Severe COVID-19 vs control	440	2,377	0.159	0.099	2.01 (1.58-2.55)	1.2×10^{-8}
		Non-Severe COVID-19 vs control	1,041	2,377	0.107	0.099	1.08 (0.90-1.30)	0.39
		Severe COVID-19 vs Non-Severe COVID-19	440	1,041	0.159	0.107	1.90 (1.43-2.52)	1.1×10^{-5}
	Age ≥ 65	COVID-19 vs control	909	912	0.113	0.106	1.08 (0.86-1.35)	0.50
		Severe COVID-19 vs control	550	912	0.104	0.106	0.93 (0.71-1.22)	0.61
		Non-Severe COVID-19 vs control	350	912	0.129	0.106	1.30 (0.98-1.73)	0.069
		Severe COVID-19 vs Non-Severe COVID-19	550	350	0.104	0.129	0.75 (0.54-1.03)	0.074

Uncorrected *P* values are shown.

Supplementary Table 9. A replication study of the *DOCK2* variant in the pan-ancestry meta-analysis

Study	Ancestry	No. subjects		Risk allele frequency (rs60200309-A)	OR (95%CI)	*P*
		Cases	Controls			
UK Biobank	EUR	1,824	428,622	0	0.44 (0.06-3.10)	0.41
	AFR	83	9,193	0	0.22 (0.00-30.5)	0.55
	SAS	71	10,252	0.00029	4.49 (0.51-39.6)	0.18
AncestryDNA	EUR	636	72,097	N.A.	4.38 (1.10-17.5)	0.037
	AFR	91	3,605	N.A.	0.60 (0.03-11.1)	0.73
	AMR	104	7,653	N.A.	1.87 (0.83-4.23)	0.13
FinnGen	EUR	82	238,353	0	0.35 (0.01-26.9)	0.64
Geisinger	EUR	180	112,862	0	0.25 (0.00-30.0)	0.57
UPENN	AFR	67	8,738	0	0.40 (0.00-43.3)	0.70
Meta-analysis		3,138	891,375	0.00082	1.73 (0.95-3.15)	0.072

EUR; European, AFR; African, AMR; American.

We obtained the association of the *DOCK2* variant (rs60200309) from the pan-ancestry meta-analysis available at https://rgc-covid19.regeneron.com/. We obtained the meta-analysis results of the phenotype of "hospitalized COVID-19 vs COVID-19 negative or COVID-19 status unknown" with the largest case sample size. Uncorrected *P* values are shown.

Supplementary Table 10. eQTL analysis result of the variants around the *DOCK2* region

(Results are indicated in a separate Microsoft Excel file)

Supplementary Table 11. The guide RNA for *DOCK2* knockdown used in LentiCRISPR system and sgRNA sequence annotation

	sgRNA Target Sequence	Rule Set 2 score	Cutting Frequency Determination score
DOCK2 KO#1	GAGAACTACCTAGTGCGATG	0.722	94
DOCK2 KO#2	GATGGGACTACTCTACACGA	0.712	97
DOCK2 KO#3	GCCAATCCTAAGAACGCTGA	0.633	94

Rule Set 2 score is used to calculate the on-target efficacy, and Cutting Frequency Determination (CFD) score is used to calculate the potential off-target efficacy.

Supplementary Table 12. The primers and probes used for evaluation of lung cytokine expression profile in SARS-CoV-2 infected Syrian hamster

Gene	Primer	Sequence(5'→3')
Ifna	Forward	CTGCCTCAGGCTCCTAACCTCAGGAACAAT
	Reverse	GTGGTGATGAGCTATTGGTCGA
	Probe	GGGAAAGTCTCATTTCTTTCAGGAG
Ifnb	Forward	TTCAACAACACTTTCTCCAGCACTGGGTGG
	Reverse	CATCCAAGAGATGCTCCAGAATATC
	Probe	CCTCTGGCACTTCCTTTAGGG
Ifng	Forward	CAGAGGAGCATAGACACCATCAAGGCAGAC
	Reverse	CCTTGAAGGACAACCAGGC
	Probe	CCTGAAGGTCATTTACCGGAATC
Il6	Forward	ACAAAGCCAGAGTCATTCAGAGCACCATCA
	Reverse	GTGACGAACAATGTACAAGATAACAA
	Probe	CGTGACTATTTTATCTGGACCCTTTA
Ccl3	Forward	CAGATTTCCCGCAAATTCATCGCCGACT
	Reverse	CCTCCTGCTGCTTCTTCTATGG
	Probe	CGGTTTCTCTTGGTTAGGAAAATGA
Ccl5	Forward	CACTCCCTGCTGCTTTGACTACCTCTCCTT
	Reverse	CGCTCCTTCATCTGCCTCC
	Probe	CTTCGGGTGACAAAAACGACTG
Ip-10	Forward	AAGGAATCCCTCTTTCAAGGACAGTCCGCT
	Reverse	CATCCTGCTGAGTCTGAGTGG
	Probe	CCCAAGGCCCTCGGTTTTAC
Gapdh	Forward	AGCCTTGACTGTGCCTTTGAACTTGCCA
	Reverse	CCTTCATTGACCTCAACTACATGG
	Probe	GCTCCTGGAAGATGGTGATGG
