# Supplementary material for: DOCK2 is involved in the host genetics and biology of severe COVID-19
Source: Nature. 2022 Aug 8;609(7928):754–60. doi: 10.1038/s41586-022-05163-5 (PMC9492544; doi:10.1038/s41586-022-05163-5)
Supplement: Supplementary file 2 — Reporting Summary [file 41586_2022_5163_MOESM2_ESM.pdf]

nature portfolio

Corresponding author(s): Yukinori Okada, Koichi Fukunaga

Last updated by author(s): Jul 28, 2022

Reporting Summary

Nature Portfolio wishes to improve the reproducibility of the work that we publish. This form provides structure for consistency and transparency in reporting. For further information on Nature Portfolio policies, see our Editorial Policies and the Editorial Policy Checklist.

Statistics

For all statistical analyses, confirm that the following items are present in the figure legend, table legend, main text, or Methods section.

n/a	Confirmed	
☐	☒	The exact sample size (*n*) for each experimental group/condition, given as a discrete number and unit of measurement
☐	☒	A statement on whether measurements were taken from distinct samples or whether the same sample was measured repeatedly
☐	☒	The statistical test(s) used AND whether they are one- or two-sided<br>*Only common tests should be described solely by name; describe more complex techniques in the Methods section.*
☐	☒	A description of all covariates tested
☐	☒	A description of any assumptions or corrections, such as tests of normality and adjustment for multiple comparisons
☐	☒	A full description of the statistical parameters including central tendency (e.g. means) or other basic estimates (e.g. regression coefficient) AND variation (e.g. standard deviation) or associated estimates of uncertainty (e.g. confidence intervals)
☐	☒	For null hypothesis testing, the test statistic (e.g. *F*, *t*, *r*) with confidence intervals, effect sizes, degrees of freedom and *P* value noted<br>*Give P values as exact values whenever suitable.*
☒	☐	For Bayesian analysis, information on the choice of priors and Markov chain Monte Carlo settings
☒	☐	For hierarchical and complex designs, identification of the appropriate level for tests and full reporting of outcomes
☐	☒	Estimates of effect sizes (e.g. Cohen's *d*, Pearson's *r*), indicating how they were calculated

Our web collection on statistics for biologists contains articles on many of the points above.

Software and code

Policy information about availability of computer code

Data collection	No software was used in data collection.
Data analysis	We used publicly available software for the data analysis (plink 1.9 and 2.0, SHAPEIT2 and 4, Minimac4, DEEP*HLA, TwoSampleMR (v.0.5.5), Trimmomatic (v0.39), STAR (v2.7.9a), RSEM (v1.3.3), eCAVIAR, Cell Ranger 5.0.0, dropEst (v0.8.6), Seurat (v3.2.2 and v4.0.2), Scrublet (v0.2.1), Nebulosa (v.1.0.0), edgeR (v3.32.0), WGCNA (v1.69), scran (v1.18.5), Clusterprofiler (v3.14.3), R (3.6.3 and 4.0.2)).

For manuscripts utilizing custom algorithms or software that are central to the research but not yet described in published literature, software must be made available to editors and reviewers. We strongly encourage code deposition in a community repository (e.g. GitHub). See the Nature Portfolio guidelines for submitting code & software for further information.

Data

Policy information about availability of data

All manuscripts must include a data availability statement. This statement should provide the following information, where applicable:

- Accession codes, unique identifiers, or web links for publicly available datasets
- A description of any restrictions on data availability
- For clinical datasets or third party data, please ensure that the statement adheres to our policy

GWAS summary statistics and processed count matrices of bulk RNA-seq are deposited at the National Bioscience Database Center (NBDC) Human Database with the accession code hum0343 without restriction (https://humandbs.biosciencedbc.jp/en/hum0343-latest). Raw sequencing data of scRNA-seq are available under controlled access at the Japanese Genotype-phenotype Archive (JGA) with accession codes JGAS000543/JGAD000662 for general research use (https://ddbj.nig.ac.jp/resource/jga-study/JGAS000543), which can be accessed through application at the NBDC with the accession code hum0197 (https://humandbs.biosciencedbc.jp/en/hum0197-latest). GWAS genotype data of the COVID-19 cases are available under controlled access at European Genome-Phenome

Archive (EGA) with the accession code EGAS00001006284 for general research use (https://ega-archive.org/studies/EGAS00001006284). GWAS genotype data of the controls collected at Osaka University and the affiliated medical institutes (n=2,380) are available under controlled access at EGA with the accession code EGAS00001006423 for the use as the controls (https://ega-archive.org/studies/EGAS00001006423). GWAS genotype data of the controls collected at University of Tsukuba (n=909) cannot be deposited since no consent was obtained for deposition in a public repository, but these data are available upon request (contact: Prof. Nobuyuki Hizawa; nhizawa@md.tsukuba.ac.jp) for the use as controls in research of inflammatory lung diseases.

Field-specific reporting

Please select the one below that is the best fit for your research. If you are not sure, read the appropriate sections before making your selection.

☒ Life sciences ☐ Behavioural & social sciences ☐ Ecological, evolutionary & environmental sciences

For a reference copy of the document with all sections, see nature.com/documents/nr-reporting-summary-flat.pdf

Life sciences study design

All studies must disclose on these points even when the disclosure is negative.

Sample size	We recruited 2,520 COVID-19 cases who required hospitalization from April 2020 to January 2021 (the 1st to 3rd pandemic waves in Japan) from >100 hospitals participating in Japan COVID-19 Task Force. 3,341 control subjects were collected as general Japanese populations at Osaka University and affiliated institutes. All sample size in GWASs in this study is summarized in Table 1, Supplementary Table2 and 8. Among COVID-19 cases, we enrolled 475 cases for bulk RNA-seq analysis and qPCR-based DE analysis. All sample size in bulk RNA-seq analysis in this study is summarized in Supplementary Table2. We recruited 30 severe COVID-19 cases and 31 healthy controls for PBMC scRNA-seq analysis. We recruited 19 healthy controls for evaluation of biological impacts of DOCK2 downregulation using primary cells. We obtained the samples of lung and hilar lymph node from autopsied cadaver died from COVID-19 pneumonia (N=3), non-COVID-19 pneumonia (N=2) and lung and lymph node tissue section surgically resected due to lung cancer for control sample (N=2).
Data exclusions	We excluded samples with low genotyping call rate, samples in close genetic relation, and ancestry outliers of East Asian population in GWAS analysis. We excluded PCA outliers of gene expression in bulk RNA-seq analysis.
Replication	We enrolled 1,243 severe COVID-19 cases collected from February 2021 to September 2021 (the 4th to 5th pandemic waves in Japan) through Japan COVID-19 Task Force and 3,769 controls as general Japanese populations at Osaka University Graduate School of Medicine, affiliated institutes and the Biobank Japan Project. We replicated an age-specific nominal risk of the DOCK2 variant (rs60200309) in the younger COVID-19 cases (OR=1.28, 95%CI=1.02-1.61, P=0.033). We also obtained the association of the rs60200309 from the pan-ancestry meta-analysis available at https://rgc-covid19.regeneron.com/. We observed the same directional effect with a marginal association signal (OR=1.73, 95%CI=0.95-3.15, P=0.072).
Randomization	We did not need to use randomization in this study because this is a genotype-phenotype association study. All the samples with available accessibility to genotype and phenotype data were included in the analysis.
Blinding	We did not apply blinding of the samples because this is a genotype-phenotype association study and no intervention was conducted in our study.

Reporting for specific materials, systems and methods

We require information from authors about some types of materials, experimental systems and methods used in many studies. Here, indicate whether each material, system or method listed is relevant to your study. If you are not sure if a list item applies to your research, read the appropriate section before selecting a response.

Materials & experimental systems

n/a	Involved in the study	
☐	☒	Antibodies
☐	☒	Eukaryotic cell lines
☒	☐	Palaeontology and archaeology
☐	☒	Animals and other organisms
☐	☒	Human research participants
☒	☐	Clinical data
☒	☐	Dual use research of concern

Methods

n/a	Involved in the study	
☒	☐	ChIP-seq
☒	☐	Flow cytometry
☒	☐	MRI-based neuroimaging

Antibodies

Antibodies used	Anti-DOCK2 rabbit polyclonal antibody was originally raised using affinity-purified DOCK2 c-terminus antigen in a previous study (Biochim Biophys Acta. 1999; 1452:179-187).<br>anti-DOCK2; Abcam#ab124838<br>anti-b-actin; Sigma#A5441<br>anti-CD68; Abcam#ab12512

Validation	Anti-DOCK2 rabbit polyclonal antibody was validated for IHC and western blotting (Blood .2002;100(12):3968-74., Biochem Biophys Res Commun. 2010 Apr 23;395(1):111-5.).<br>Anti-DOCK2 antibody is validated for WB and IHC (Abcam).<br>Anti-β-actin antibody is validated for WB and IF (Sigma).<br>Anti-CD68 antibody is validated for IHC (Abcam).

Eukaryotic cell lines

Policy information about cell lines

Cell line source(s)	THP1-Blue ISG cells (human THP-1 monocyte cell line by stable integration of an interferon regulatory factor (IRF)-inducible SEAP reporter construct)
Authentication	None, but used for experiments within two months after obtaining from the vendor, Invivogen.
Mycoplasma contamination	No mycoplasma
Commonly misidentified lines<br>(See ICLAC register)	None.

Animals and other organisms

Policy information about studies involving animals; ARRIVE guidelines recommended for reporting animal research

Laboratory animals	Syrian hamsters were purchased from CLEA Japan, Inc. Tokyo, Japan. Six-week-old male Syrian hamsters were maintained in the biological safety level 3 experimental animal facility of the Department of Veterinary Medicine, Kitasato University. Animals were cared for according to the Guidelines for Animal Experiments of Kitasato University and the National Institutes of Health Guide for the Care and Use of Laboratory Animals.
Wild animals	Not used in this study.
Field-collected samples	Not used in this study.
Ethics oversight	The animal experimentation protocol was approved by the President of Kitasato University through the judgment of the Institutional Animal Care and Use Committee of Kitasato University (approval no. 21-007).

Note that full information on the approval of the study protocol must also be provided in the manuscript.

Human research participants

Policy information about studies involving human research participants

Population characteristics	COVID-19 cases in the GWAS are of East Asian ancestry, the mean age was 56, 64% were male, and all of them were tested positive for PCR test results. Controls in the GWAS are of East Asian ancestry, the mean age was 53, 48% were male. Mean age of COVID-19 cases in the bulk RNA-seq analysis was 60, 68% were male. Details of the characteristics of the study participants in the GWAS, bulk RNA-seq analysis and replication analysis are summarized in Supplementary Table2.
Recruitment	We enrolled the hospitalized cases diagnosed as COVID-19 by physicians using the clinical manifestation and PCR test results, who were recruited from April 2020 to January 2021 (the 1st to 3rd pandemic waves in Japan) at any of the >100 the affiliated hospitals participating to Japan COVID-19 Task Force. All control participants in GWAS were recruited at Osaka University or related institutions. We incorporated 475 COVID-19 cases collected at the core medical institutes of Japan COVID-19 Task Force and included in the GWAS for bulk RNA-seq analysis and qPCR-based DE analysis. We enrolled severe COVID-19 cases and healthy controls for PBMC scRNA-seq analysis at Osaka University. We recruited healthy controls for evaluation of biological impacts of DOCK2 downregulation using primary cells at Osaka University. We obtained the samples of lung and hilar lymph node from autopsied cadaver died from COVID-19 pneumonia, non-COVID-19 pneumonia and lung and lymph node tissue section surgically resected due to lung cancer for control sample through Japan COVID-19 Task Force.
Ethics oversight	This study was approved by the ethical committees of Keio University School of Medicine, Osaka University Graduate School of Medicine, and affiliated institutes.

Note that full information on the approval of the study protocol must also be provided in the manuscript.
